# PADI4 has genetic susceptibility to gastric carcinoma and upregulates CXCR2, KRT14 and TNF-α expression levels

**DOI:** 10.18632/oncotarget.11398

**Published:** 2016-08-19

**Authors:** Yabing Zheng, Gang Zhao, Bing Xu, Chunyan Liu, Chang Li, Xiaoqian Zhang, Xiaotian Chang

**Affiliations:** ^1^ Medical Research Center of Shandong Provincial Qianfoshan Hospital, Shandong University, Jinan, Shandong, P. R. China; ^2^ Emergency Surgery Department of Shandong Provincial Qianfoshan Hospital, Shandong University, Jinan, Shandong, P. R. China; ^3^ Pathological Department of Tengzhou People's Central Hospital, Tengzhou, Shandong, P. R. China; ^4^ Clinical Laboratory of PKUCare Luzhong Hospital, Zibo, Shandong, P. R. China

**Keywords:** peptidyl deiminase (PAD), peptidyl deiminase isoform 4 (PADI4), CXCR2, KRT14, TNF-α

## Abstract

PADI4 (peptidyl deiminase isoform 4) is overexpressed in many tumor tissues and converts arginine residues to citrulline residues. This study used an Illumina SNP microarray and a TaqMan assay to determine the possible association of the PADI4 gene with various tumor risks. Both genotyping methods demonstrated significant associations between the tag SNPs rs1635566 and rs882537 in the PADI4 locus with gastric carcinoma in two independent cohorts. Based on this genotyping result, we used the Cancer Pathway Finder, p53 Signaling, Signal Transduction and Tumor Metastasis PCR arrays to investigate the tumorigenic pathway of PADI4 in MNK-45 cells derived from gastric carcinoma. We detected significantly decreased expression levels of CXCR2, KRT14 and TNF-α in MNK-45 cells that were treated with anti-PADI4 siRNA. We also detected increased expression of these three genes in MNK-45 cells transfected with a pcDNA3.1 plasmid overexpressing PADI4. A highly similar result was also obtained for SGC 7901 cells, which also originate from gastric carcinoma. Our result indicates that the PADI4 gene has genetic susceptibility in gastric carcinoma. PADI4 contributes to gastric tumorigenesis by upregulating CXCR2, KRT14 and TNF-α expression, which are well known to activate angiogenesis, cell proliferation, cell migration and the immune microenvironment in tumors.

## INTRODUCTION

Peptidyl arginine deiminase (PAD) catalyzes the conversion of arginine residues to citrulline residues in the presence of excess calcium. This enzymatic activity is referred as citrullination or, alternatively, as deimination. PAD-mediated post-translational citrullination processes play important roles in protein function and structural stability and, therefore, significantly affect physiological and pathological processes [1, 2]. The roles of PAD and citrullination in tumorigenesis have continued to garner increased interest [3, 4]. Five mammalian PAD family members (PAD or PADI 1-4 and 6) are all encoded by a cluster of genes on chromosome 1p36.13 [5]. Increasing evidence suggests that PADI4 (peptidyl deiminase isoform 4) is involved in tumor progression [6–8]. We and others demonstrated marked overexpression of PADI4 in many human cancers, including cervical squamous cell carcinoma, gastric carcinoma, lung cancer, ovarian serous papillary adenocarcinoma and papillary carcinoma of the thyroid [9, 10]. We and others also detected a significant increase in the levels of PADI4 and citrullinated antithrombin in the blood of patients with tumors [10]. In addition, we found that elevated PADI4 levels are positively correlated with the pathological classification of esophageal squamous cell carcinoma and that the PADI4 gene has a valid susceptibility to the risk of esophageal carcinoma [11, 12]. Moreover, we reported that the expression level of PADI4 was related to the patient's age and the histopathological classification of ovarian serous adenocarcinoma. Estrogen can stimulate the expression of PADI4 in ovarian tumor cells [13]. Recently, we found that PADI4 can stimulate the proliferation, apoptosis, invasion and migration of the ovarian cancer cell line A2480 via p53 signaling [14].

To gain further insight into its tumorigenic mechanism, we investigated whether common polymorphisms in the PADI4 region are associated with various cancer risks using a SNP microarray. The results were verified by the TaqMan genotyping method in independent cohorts. We aimed to determine the tumor types to which the PADI4 gene has valid susceptibility. Based on the genetics data, the study continued to explore the effect of PADI4 on the tumorigenic process and determine the signaling pathway by which PADI4 contributes to tumorigenesis.

## RESULTS

### Genotyping SNPs located in the PADI4 locus

We genotyped 59 SNPs in the PADI4 locus of various tumor samples and controls using an Illumina 384-SNP VeraCode microarray. All of the SNPs yielded usable genotyping data, and the study sample success rate was 98.9%. Differences in the allele and genotype frequencies between the cancer and control samples were compared. The genotypic frequency of the SNPs at rs882537, rs1748034, rs1748032, rs1635566 and rs1635584 were significantly different between the gastric carcinoma cohort and the healthy control cohort. In addition, the allelic frequency and genotypic frequency of the SNP at rs1635586 were significantly different in patients with either breast cancer or esophageal carcinoma compared to the control subjects. The allelic frequency of the SNP at rs1635564 was significantly different between the rectal carcinoma cohorts and the control cohort. Table 1 presents the allele frequencies, genotype frequencies and p-values after the statistical analysis of the above-mentioned SNPs. Other SNPs located in the PADI4 gene did not demonstrate a significant difference in either the allelic or genotypic frequencies between the tumors and control samples.

**Table 1:** Genotyping result of illumina microarray (control n=384)

Locus Name	Breast cancer (n=281)	Cervical carcinoma (n=197)	esophageal carcinoma (n=221)	gastric carcinoma (n=308)	liver cancer (n=202)	ovarian cancer (n=157)	rectal carcinoma (n=101)
**rs882537**							
Allele	A G	A G	A G	A G	A G	A G	A G
allele case (frequency)	212(0.385) 338(0.615)	119(0.363) 209(0.637)	147(0.362) 259(0.638)	235(0.390) 367(0.610)	138(0.347) 260(0.653)	112(0.366) 194(0.634)	64(0.372) 108(0.628)
allele control (frequency)	275(0.370) 469(0.630)	275(0.370) 469(0.630)	275(0.370) 469(0.630)	275(0.370) 469(0.630)	275(0.370) 469(0.630)	275(0.370) 469(0.630)	275(0.370) 469(0.630)
Odds Ratio [%95 CI]	1.069693 [0.852240 ~1.342632]	0.971048 [0.741383 ~1.271859]	0.967961 [0.752907 ~1.244440]	1.092049 [0.875248 ~1.362551]	0.905203 [0.701803 ~1.167553]	0.984592 [0.747004 ~1.297746]	1.010640 [0.717077 ~1.424384]
Fisher's p value	0.561209	0.831035	0.799479	0.435459	0.443042	0.91225	0.9518
Genotype	A/A A/G G/G	A/A A/G G/G	A/A A/G G/G	A/A A/G G/G	A/A A/G G/G	A/A A/G G/G	A/A A/G G/G
genotype case (frequency)	35(0.127) 142(0.516) 98(0.356)	17(0.104) 85(0.518) 62(0.378)	21(0.103) 105(0.517) 77(0.379)	32(0.106) 171(0.568) 98(0.326)	29(0.146) 80(0.402) 90(0.452)	20(0.131) 72(0.471) 61(0.399)	8(0.093) 48(0.558) 30(0.349)
genotype control (frequency)	52(0.140) 171(0.460) 149(0.401)	52(0.140) 171(0.460) 149(0.401)	52(0.140) 171(0.460) 149(0.401)	52(0.140) 171(0.460) 149(0.401)	52(0.140) 171(0.460) 149(0.401)	52(0.140) 171(0.460) 149(0.401)	52(0.140) 171(0.460) 149(0.401)
Fisher's p value	0.360218	0.346713	0.297829	0.019414	0.398237	0.954194	0.21665
HWE for case (Fisher's p)	0.135942	0.12136	0.088194	0.000789	0.112263	0.86261	0.071416
HWE for case (Fisher's p)	0.793448	0.793448	0.793448	0.793448	0.793448	0.793448	0.793448
**rs1748034**							
Allele	A C	A C	A C	A C	A C	A C	A C
allele case (frequency)	209(0.377) 345(0.623)	116(0.354) 212(0.646)	140(0.337) 276(0.663)	225(0.371) 381(0.629)	138(0.343) 264(0.657)	108(0.353) 198(0.647)	64(0.372) 108(0.628)
allele control (frequency)	262(0.350) 486(0.650)	262(0.350) 486(0.650)	262(0.350) 486(0.650)	262(0.350) 486(0.650)	262(0.350) 486(0.650)	262(0.350) 486(0.650)	262(0.350) 486(0.650)
Odds Ratio [%95 CI]	1.123731 [0.894435 ~1.411808]	1.014979 [0.773521 ~1.331810]	0.940923 [0.730716 ~1.211600]	1.095450 [0.876496 ~1.369100]	0.969639 [0.751491 ~1.251114]	1.011797 [0.765879 ~1.336678]	1.099237 [0.779477 ~1.550169]
Fisher's p value	0.316371	0.914581	0.636887	0.422938	0.812584	0.93421	0.589495
Genotype	A/A A/C C/C	A/A A/C C/C	A/A A/C C/C	A/A A/C C/C	A/A A/C C/C	A/A A/C C/C	A/A A/C C/C
genotype case (frequency)	29(0.105) 151(0.545) 97(0.350)	14(0.085) 88(0.537) 62(0.378)	16(0.077) 108(0.519) 84(0.404)	21(0.069) 183(0.604) 99(0.327)	24(0.119) 90(0.448) 87(0.433)	14(0.092) 80(0.523) 59(0.386)	8(0.093) 48(0.558) 30(0.349)
genotype control (frequency)	43(0.115) 176(0.471) 155(0.414)	43(0.115) 176(0.471) 155(0.414)	43(0.115) 176(0.471) 155(0.414)	43(0.115) 176(0.471) 155(0.414)	43(0.115) 176(0.471) 155(0.414)	43(0.115) 176(0.471) 155(0.414)	43(0.115) 176(0.471) 155(0.414)
Fisher's p value	0.164603	0.31208	0.273499	0.001727	0.871793	0.500083	0.340914
HWE for case (Fisher's p)	0.007706	0.02615	0.018966	3.32E-07	0.921884	0.073379	0.071416
HWE for case (Fisher's p)	0.512169	0.512169	0.512169	0.512169	0.512169	0.512169	0.512169
**rs1748032**							
Allele	A G	A G	A G	A G	A G	A G	A G
allele case (frequency)	331(0.600) 221(0.400)	205(0.629) 121(0.371)	262(0.639) 148(0.361)	368(0.609) 236(0.391)	257(0.642) 143(0.357)	192(0.623) 116(0.377)	104(0.605) 68(0.395)
allele control (frequency)	475(0.637) 271(0.363)	475(0.637) 271(0.363)	475(0.637) 271(0.363)	475(0.637) 271(0.363)	475(0.637) 271(0.363)	475(0.637) 271(0.363)	475(0.637) 271(0.363)
Odds Ratio [%95 CI]	0.854499 [0.681384 ~1.071596]	0.966594 [0.738081 ~1.265856]	1.009986 [0.785977 ~1.297839]	0.889634 [0.713010 ~1.110011]	1.025352 [0.796048 ~1.320706]	0.944319 [0.717587 ~1.242691]	0.872570 [0.620982 ~1.226087]
Fisher's p value	0.173334	0.804995	0.938099	0.300317	0.846299	0.682565	0.432009
Genotype	A/A A/G G/G	A/A A/G G/G	A/A A/G G/G	A/A A/G G/G	A/A A/G G/G	A/A A/G G/G	A/A A/G G/G
genotype case (frequency)	96(0.348) 139(0.504) 41(0.149)	61(0.374) 83(0.509) 19(0.117)	84(0.410) 94(0.459) 27(0.132)	99(0.328) 170(0.563) 33(0.109)	87(0.435) 83(0.415) 30(0.150)	60(0.390) 72(0.468) 22(0.143)	31(0.360) 42(0.488) 13(0.151)
genotype control (frequency)	154(0.413) 167(0.448) 52(0.139)	154(0.413) 167(0.448) 52(0.139)	154(0.413) 167(0.448) 52(0.139)	154(0.413) 167(0.448) 52(0.139)	154(0.413) 167(0.448) 52(0.139)	154(0.413) 167(0.448) 52(0.139)	154(0.413) 167(0.448) 52(0.139)
Fisher's p value	0.236323	0.409016	0.9537	0.01196	0.750852	0.883049	0.671026
HWE for case (Fisher's p)	0.416619	0.246259	0.930562	0.001544	0.171856	0.957344	0.842021
HWE for case (Fisher's p)	0.534238	0.534238	0.534238	0.534238	0.534238	0.534238	0.534238
**rs1635566**							
Allele	A G	A G	A G	A G	A G	A G	A G
allele case (frequency)	161(0.387) 255(0.613)	113(0.345) 215(0.655)	113(0.360) 201(0.640)	187(0.374) 313(0.626)	121(0.348) 227(0.652)	107(0.354) 195(0.646)	62(0.369) 106(0.631)
allele control (frequency)	207(0.366) 359(0.634)	207(0.366) 359(0.634)	207(0.366) 359(0.634)	207(0.366) 359(0.634)	207(0.366) 359(0.634)	207(0.366) 359(0.634)	207(0.366) 359(0.634)
Odds Ratio [%95 CI]	1.094989 [0.843345 ~1.421720]	0.911516 [0.685595 ~1.211883]	0.975004 [0.731753 ~1.299117]	1.036147 [0.807638 ~1.329310]	0.924450 [0.699270 ~1.222143]	0.951641 [0.711145 ~1.273469]	1.014402 [0.709837 ~1.449643]
Fisher's p value	0.495774	0.523744	0.862756	0.779993	0.581252	0.738764	0.937434
Genotype	A/A A/G G/G	A/A A/G G/G	A/A A/G G/G	A/A A/G G/G	A/A A/G G/G	A/A A/G G/G	A/A A/G G/G
genotype case (frequency)	30(0.144) 101(0.486) 77(0.370)	16(0.098) 81(0.494) 67(0.409)	18(0.115) 77(0.490) 62(0.395)	23(0.092) 141(0.564) 86(0.344)	24(0.138) 73(0.420) 77(0.443)	17(0.113) 73(0.483) 61(0.404)	9(0.107) 44(0.524) 31(0.369)
genotype control (frequency)	40(0.141) 127(0.449) 116(0.410)	40(0.141) 127(0.449) 116(0.410)	40(0.141) 127(0.449) 116(0.410)	40(0.141) 127(0.449) 116(0.410)	40(0.141) 127(0.449) 116(0.410)	40(0.141) 127(0.449) 116(0.410)	40(0.141) 27(0.449) 116(0.410)
Fisher's p value	0.657844	0.359992	0.612557	0.020706	0.782344	0.642277	0.444875
HWE for case (Fisher's p)	0.735687	0.230923	0.41902	0.001232	0.321857	0.486751	0.252864
HWE for case (Fisher's p)	0.582126	0.582126	0.582126	0.582126	0.582126	0.582126	0.582126
**rs1635584**							
Allele	A G	A G	A G	A G	A G	A G	A G
allele case (frequency)	252(0.603) 166(0.397)	195(0.613) 123(0.387)	197(0.627) 117(0.373)	309(0.623) 187(0.377)	228(0.655) 120(0.345)	189(0.622) 115(0.378)	97(0.606) 63(0.394)
allele control (frequency)	354(0.628) 210(0.372)	354(0.628) 210(0.372)	354(0.628) 210(0.372)	354(0.628) 210(0.372)	354(0.628) 210(0.372)	354(0.628) 210(0.372)	354(0.628) 210(0.372)
Odds Ratio [%95 CI]	0.900551 [0.694462 ~1.167799]	0.940471 [0.708677 ~1.248081]	0.998841 [0.750807 ~1.328815]	0.980241 [0.763992 ~1.257699]	1.127119 [0.852455 ~1.490279]	0.974945 [0.731062 ~1.300187]	0.913371 [0.637125 ~1.309393]
Fisher's p value	0.429479	0.67077	0.993647	0.875301	0.400987	0.862848	0.621906
Genotype	A/A A/G G/G	A/A A/G G/G	A/A A/G G/G	A/A A/G G/G	A/A A/G G/G	A/A A/G G/G	A/A A/G G/G
genotype case (frequency)	76(0.364) 100(0.478) 33(0.158)	55(0.346) 85(0.535) 19(0.119)	60(0.382) 77(0.490) 20(0.127)	85(0.343) 139(0.560) 24(0.097)	78(0.448) 72(0.414) 24(0.138)	58(0.382) 73(0.480) 21(0.138)	29(0.362) 39(0.487) 12(0.150)
genotype control (frequency)	113(0.401) 128(0.454) 41(0.145)	113(0.401) 128(0.454) 41(0.145)	113(0.401) 128(0.454) 41(0.145)	113(0.401) 128(0.454) 41(0.145)	113(0.401) 128(0.454) 41(0.145)	113(0.401) 128(0.454) 41(0.145)	113(0.401) 128(0.454) 41(0.145)
Fisher's p value	0.701485	0.262852	0.736532	0.035069	0.601152	0.871057	0.821404
HWE for case (Fisher's p)	0.991177	0.109477	0.539408	0.002364	0.266702	0.795472	0.85025
HWE for case (Fisher's p)	0.627543	0.627543	0.627543	0.627543	0.627543	0.627543	0.627543
**rs1635586**							
Allele	A G	A G	A G	A G	A G	A G	A G
allele case (frequency)	96(0.229) 324(0.771)	61(0.186) 267(0.814)	75(0.237) 241(0.763)	100(0.165) 506(0.835)	70(0.201) 278 (0.799)	57(0.185) 251(0.815)	26(0.151) 146(0.849)
allele control (frequency)	127(0.170) 621(0.830)	127(0.170) 621(0.830)	127(0.170) 621(0.830)	127(0.170) 621(0.830)	127(0.170) 621 (0.830)	127(0.170) 621(0.830)	127(0.170) 621(0.830)
Odds Ratio [%95 CI]	1.448819 [1.076356 ~1.950169]	1.117137 [0.797261 ~1.565354]	1.521711 [1.102931 ~2.099500]	0.966356 [0.725168~ 1.287763]	1.231236 [0.890275~ 1.702779]	1.110424 [0.786298~ 1.568162]	0.870780 [0.550367~ 1.377731]
Fisher's p value	0.014201	0.519743	0.010275	0.815293	0.208163	0.551903	0.554242
Genotype	A/A A/G G/G	A/A A/G G/G	A/A A/G G/G	A/A A/G G/G	A/A A/G G/G	A/A A/G G/G	A/A A/G G/G
genotype case (frequency)	11(0.052) 74(0.352) 125(0.595)	6(0.037) 49(0.299) 109(0.665)	5(0.032) 65(0.411) 88(0.557)	9(0.030) 82(0.271) 212(0.700)	7(0.040) 56 (0.322) 111(0.638)	6(0.039) 45(0.292) 103(0.669)	1(0.012) 24(0.279) 61(0.709)
genotype control (frequency)	10(0.027) 107(0.286) 257(0.687)	10(0.027) 107(0.286) 257(0.687)	10(0.027) 107(0.286) 257(0.687)	10(0.027) 107 (0.286) 257 (0.687)	10(0.027) 107 (0.286) 257 (0.687)	10(0.027) 107(0.286) 257(0.687)	10(0.027) 107(0.286) 257(0.687)
Fisher's p value	0.04737	0.771033	0.015107	0.889725	0.441476	0.738705	0.694467
HWE for case (Fisher's p)	0.991079	0.865763	0.08655	0.754779	0.984858	0.698239	0.417361
HWE for case (Fisher's p)	0.774391	0.774391	0.774391	0.774391	0.774391	0.774391	0.774391
**rs1635564**							
Allele	A C	A C	A C	A C	A C	A C	A C
allele case (frequency)	108(0.197) 440(0.803)	71(0.219) 253(0.781)	87(0.209) 329(0.791)	124(0.205) 482(0.795)	86(0.214) 316(0.786)	63(0.205) 245(0.795)	24(0.140) 148(0.860)
allele control (frequency)	162(0.217) 584(0.783)	162(0.217) 584(0.783)	162(0.217) 584(0.783)	162(0.217) 584(0.783)	162(0.217) 584(0.783)	162(0.217) 584(0.783)	162(0.217) 584(0.783)
Odds Ratio [%95 CI]	0.884848 [0.673373 ~1.162739]	1.011662 [0.737881~ 1.387027]	0.953282 [0.710835~ 1.278420]	0.927411 [0.712841~ 1.206570]	0.981091 [0.730338~ 1.317937]	0.926984 [0.668412~ 1.285583]	0.584585 [0.367163~ 0.930757]
Fisher's p value	0.379856	0.942591	0.749316	0.574569	0.899128	0.64951	0.022463
Genotype	A/A A/C C/C	A/A A/C C/C	A/A A/C C/C	A/A A/C C/C	A/A A/C C/C	A/A A/C C/C	A/A A/C C/C
genotype case (frequency)	12(0.044) 84(0.307) 178(0.650)	9(0.056) 53(0.327) 100(0.617)	7(0.034) 73(0.351) 128(0.615)	16(0.053) 92(0.304) 195(0.644)	9(0.045) 68(0.338) 124(0.617)	10(0.065) 43(0.279) 101(0.656)	1(0.012) 22(0.256) 63(0.733)
genotype control (frequency)	17(0.046) 128(0.343) 228(0.611)	17(0.046) 128(0.343) 228(0.611)	17(0.046) 28(0.343) 228(0.611)	17(0.046) 128(0.343) 228(0.611)	17(0.046) 128(0.343) 228(0.611)	17(0.046) 128(0.343) 228(0.611)	17(0.046) 128(0.343) 228(0.611)
Fisher's p value	0.598292	0.850607	0.784008	0.532454	0.991201	0.284056	0.070487
HWE for case (Fisher's p)	0.604255	0.575182	0.379308	0.242208	0.933481	0.078206	0.544735
HWE for case (Fisher's p)	0.85744	0.85744	0.85744	0.85744	0.85744	0.85744	0.85744

The Illumina microarray result was verified in a dependent cohort of gastric cancer patients using the TaqMan genotyping method. All 5 SNPs (rs41265997, rs1635566, rs882537, rs1635586 and rs1748034) yielded genotypic data, and the study sample success rate was 99%. Allelic frequencies and gene frequencies of these SNPs did not deviate from Hardy-Weinberg equilibrium (HWE) in either the tumor or the control samples. The genotyping did not detect the single nucleotide polymorphism at rs41265997 in the Chinese population tested. The allele frequency (odds ratio=3.827392, 95% CI=[1.262104–11.606753], p=0.010243) and gene frequency (p=0.028771) for rs1635566 demonstrated a statistically significant association with gastric carcinoma. The SNP at rs882537 also showed significant differences with regard to the allele frequency (odds ratio=1.337557, 95%CI= [1.019987~1.754002], p=0.035307) in the gastric carcinoma cohort. The genotyping assay did not detect a significant difference in either the allelic or genotypic frequencies of rs1635586 and rs1748034 (p>0.05) between patients with gastric cancer and healthy control subjects. Following multiple-test corrections, the SNPs at rs882537 and rs1635566 still showed a significant difference with regard to the allelic frequency and genotypic frequency. These results are shown in Table 2. Both the Illumina microarray assay and TaqMan assay demonstrated that the nucleotide variants at rs882537 and rs1635566 are significantly associated with gastric cancer risk.

**Table 2:** Taqman genotyping result (gastric carcinoma n= 288, control n=288)

**rs1635566**	
Allele	C(freq) T(freq)
Allele case	544(0.993) 4(0.007)
Allele control	533(0.973) 15(0.027)
Odds Ratio[%95 CI]	3.827392[1.262104~11.606753]
Fisher's p value	0.010935
Genotype	C/C(freq) C/T(freq)
Genotype case	270(0.985) 4(0.015)
Genotype control	259(0.945) 15(0.055)
Odds Ratio[%95 CI]	3.909266[1.280563~11.934097]
Fisher's p value	0.010243
H-W for cases Fisher's p value	0.903131
H-W for control Fisher's p value	0.641348
**rs882537**	
Allele	A(freq) G(freq)
Allele case	181(0.395) 277(0.605)
Allele control	149(0.328) 305(0.672)
Odds Ratio[%95 CI]	1.337557[1.019987~1.754002]
Fisher's p value	0.035307
Genotype	A/A(freq) A/G(freq) G/G(freq)
Genotype case	29(0.127) 123(0.537) 77(0.336)
Genotype control	18(0.079) 113(0.498) 96(0.423)
Fisher's p value	0.079117
H-W for cases Fisher's p value	0.061483
H-W for control Fisher's p value	0.052219
**rs1635586**	
Allele	A(freq) G(freq)
Allele case	176(0.374) 294(0.626)
Allele control	165(0.342) 317(0.658)
Odds Ratio[%95 CI]	1.150113[0.882257~1.499291]
Fisher's p value	0.301099
Genotype	A/A(freq) A/G(freq) G/G(freq)
Genotype case	29(0.123) 118(0.502) 88(0.374)
Genotype control	22(0.091) 121(0.502) 98(0.407)
Fisher's p value	0.481797
H-W for cases Fisher's p value	0.270991
H-W for control Fisher's p value	0.074184
**rs1748034**	
Allele	A(freq) C(freq)
Allele case	215(0.425) 291(0.575)
Allele control	193(0.402) 287(0.598)
Odds Ratio[%95 CI]	1.098677[0.852498~1.415946]
Fisher's p value	0.467177
Genotype	A/A(freq) A/C(freq) C/C(freq)
Genotype case	32(0.126) 151(0.597) 70(0.277)
Genotype control	32(0.133) 129(0.537) 79(0.329)
Fisher's p value	0.380914
H-W for cases Fisher's p value	0.000437
H-W for control Fisher's p value	0.067902

This genotyping study encouraged us to focus on the pathogenic mechanism of PADI4 in gastric cancer.

### Determination of the pathogenic pathway of PADI4 in the tumorigenesis of gastric carcinoma

**Figure 1:**
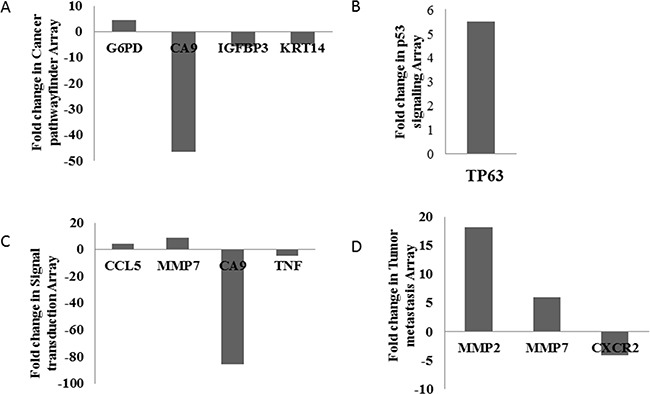
Determination of the pathogenic signaling pathway of PADI4 using a series of PCR arrays The gastric tumor cell line MNK-45 was treated with anti-PADI4 siRNA. **A.** Cancer Pathwayfinder, **B.** p53 Signaling, **C.** Signal Transduction and **D.** Tumor Metastasis PCR arrays were performed to detect any significantly altered expression levels of tumor-related genes in the treated MNK-45 cells. The fold changes were calculated and expressed as log-normalized ratios of the siRNA-treated cells/AllStar siRNA-treated cells.

MNK-45 cells were treated with anti-PADI4 siRNA. Real-time PCR was then used to examine the mRNA levels of PADI4 in the cells. The expression of PADI4 in the siRNA-treated cells was approximately 4-fold lower than that in the cells treated with AllStar siRNA (Supplementary Figure S1). This observation indicates the successful inhibition of PADI4 gene expression with anti-PADI4 siRNA. A series of Qiagen PCR array analyses were applied to examine the alternative gene expression in the treated MNK-45 cells, which allowed for the determination of the tumorigenic pathway of PADI4. Genes with at least a 4-fold change in expression were considered to be biologically significant in this study. Finally, the use of the Cancer Pathwayfinder, p53 Signaling, Signal Transduction and Tumor Metastasis PCR arrays revealed 10 genes with significantly altered expression levels in MNK-45 cells following treatment with anti-PADI4 siRNA. Significantly alterations in the expression levels of G6PD (glucose-6-phosphate dehydrogenase), CA9 (carbonic anhydrase 9), IGFBP3 (insulin like growth factor binding protein 3) and KRT14 (keratin 14) were detected by the Cancer Pathwayfinder assay; TP63 (tumor protein p63) expression was detected by the p53 Signaling Pathway array; CCL5 (C-C motif chemokine ligand 5), MMP7 (matrix metallopeptidase 7), CA9 and TNF-α (tumor necrosis factor–alpha) were detected by the Signal Transduction Pathway array; and MMP2 (matrix metallopeptidase 2), MMP7 and CXCR2 (C-X-C motif chemokine receptor 2) expression was detected by the Tumor Metastasis PCR array. The results are shown in Figure 1, and the raw data are presented in Supplementary Figure S2 and Supplementary Table S1.

**Figure 2:**
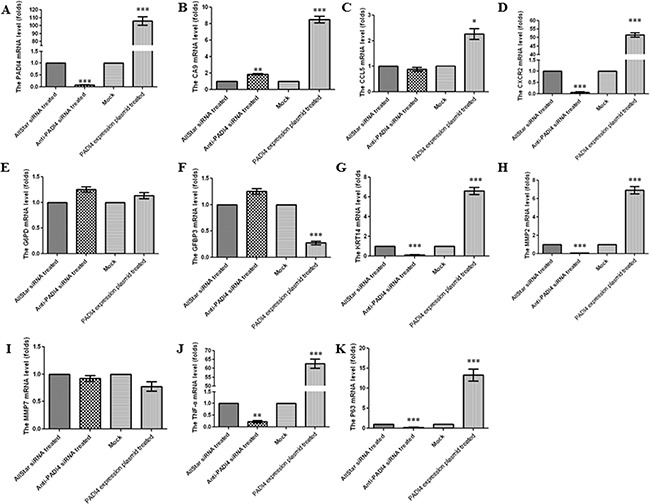
Determination of the mRNA expression levels of A PADI4, **B.** CA9, **C.** CCL5, **D.** CXCR2, **E.** G6PD, **F.** IGFBP3, **G.** KRT14, **H.** MMP2, **I.** MMP7, **J.** TNF-α and **K.** TP63 in MNK-45 cells either treated with anti-PADI4 siRNA or transfected with a PADI4-overexpressing plasmid using real-time PCR. The experiment was repeated three times. To quantify expression in the silenced PADI4 cells, the mRNA expression level of each gene in the AllStar siRNA-treated cells was set to “1”, and the expression level of the target gene in the anti-PADI4 siRNA-treated cells was normalized to the mRNA level of its corresponding gene in the AllStar siRNA-treated cells. To quantify expression in cells overexpressing PADI4, the mRNA expression level of each gene in MNK-45 cells transfected with empty pcDNA3.1 vector was set to “1”, and the expression level of the target gene in the cells transfected with PADI4-overexpressing plasmid was normalized to its corresponding gene expression in the mock cells. The expression levels are expressed as the mean±standard error of the mean. * p<0.05, ** p<0.01 and *** p<0.001.

**Figure 3:**
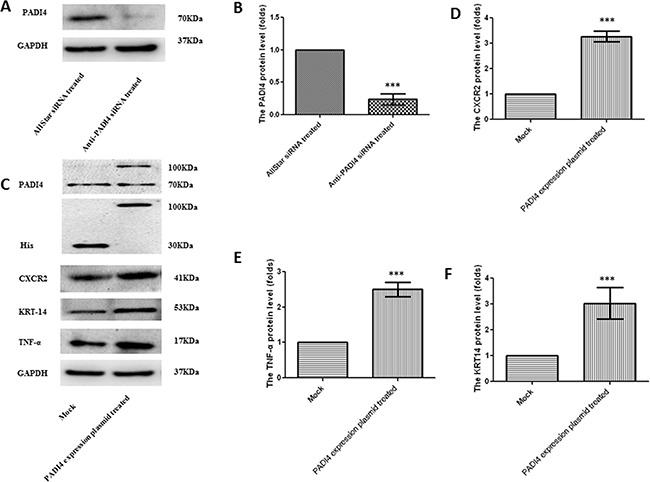
Determination of the protein expression levels of PADI4, CXCR2, KRT-14 and TNF-α in MNK-45 cells either treated with anti-PADI4 siRNA or transfected with a PADI4-overexpressing plasmid using western blot analysis **A.** The protein expression of PADI4 in the siRNA-treated MNK-45 cells. **B.** PADI4 expression was normalized to the GAPDH expression in the siRNA-treated MNK-45 cells. **C.** The protein expression of PADI4, His, CXCR2, KRT-14 and TNF-α in MNK-45 cells transfected with a recombinant pcDNA3.1 vector overexpressing PADI4. The expression of CXCR2 **D.**, KRT-14 **E.** and TNF-α **F.** were normalized to the GAPDH expression. The expression levels are expressed as the mean±standard error of the mean. *** p<0.001.

Real-time PCR analysis was used to verify the results of the PCR array in the MNK-45 cells treated with anti-PADI4 siRNA. The PADI4 mRNA levels in these cells were examined using real-time PCR, and the experiment was repeated three times. The expression of PADI4 in the siRNA-treated cells was approximately 10-fold lower than that in the cells treated with AllStar siRNA (Figure 2A). Western blot analysis also detected a nearly 5-fold decrease in the PADI4 protein expression level in treated cells (Figure 3A and 3B). These results indicate the successful inhibition of PADI4 gene expression by anti-PADI4 siRNA. The real-time analysis also detected a significant increase in the expression level of CA9 and a decrease in the expression levels of CXCR2, KRT14, MMP2, TNF-α and TP63 in anti-PADI4 siRNA-treated MNK-45 cells. No significant changes were detected in the expression levels of CCL5, G6PD, IGFBP3 and MMP7 (Figure 2B-2K). The decreased expression levels of CXCR2, KRT14 and TNF-α were consistent with the results obtained by the PCR arrays.

The results of the PCR arrays were also verified by real-time PCR using MNK-45 cells transfected with the recombinant pcDNA3.1 vector with the PADI4 gene insert. The transcription of the PADI4 gene was significantly increased by approximately 120-fold in the MNK-45 cells transfected with the PADI4-expression plasmids compared with cells transfected with mock plasmids (Figure 2A). Western blotting analysis detected a band with a molecular weight of 100 kDa with an anti-His antibody in the MNK-45 cells transfected with the recombinant overexpression vector. However, in the cells transfected with blank pcDNA3.1 vector, only a band at a molecular weight of 30 kDa was detected. Western blotting also detected PADI4 protein at molecular weights of 100 kDa and 70 kDa from the extracts of MNK-45 cells transfected with the recombinant overexpression vector, whereas PADI4 was only detected at a molecular weight of 70 kDa in the extracts of cells transfected with blank pcDNA3.1 vector. These observations indicate the successful transfection of the recombinant plasmid and the overexpression of PADI4 in MNK-45 cells. These results are shown in Figure 3C. Real-time PCR analysis detected a significant increase in the expression levels of CA9, CXCR2, KRT14, MMP2, TNF-α and TP63, as well as a considerable decrease in the expression level of IGFBP3 in PADI4-overexpressing MNK-45 cells. These experiments were repeated three times, and the results are shown in Figure 2B-2K. Western blot analysis detected an approximately 3-fold increase in the protein expression levels of CXCR2, KRT14 and TNF-α in the PADI4-overexpressing MNK-45 cells (Figure 3C-3F). The increased expression of CXCR2, KRT14 and TNF-α in the PADI4-overexpressing MNK-45 cells corresponded to the decreased expression of these targeted genes in the PADI4-silenced cells, which was detected by PCR array and real-time PCR.

**Figure 4:**
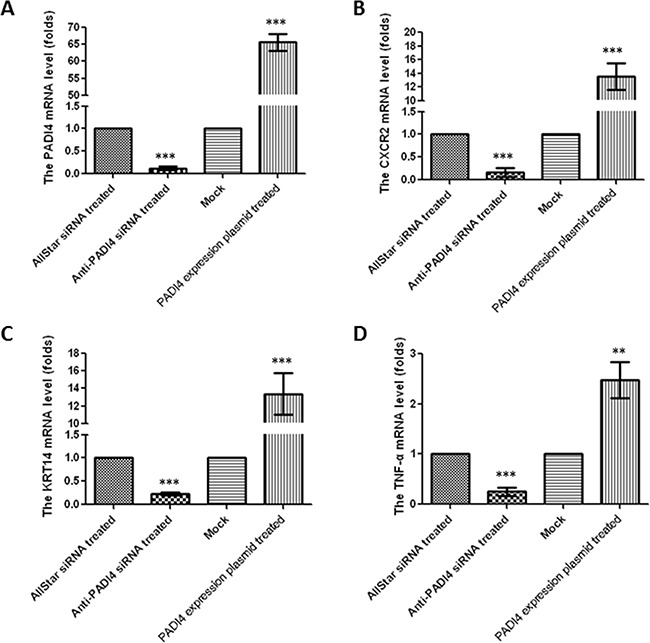
Determination of mRNA expression levels of A PADI4, **B.** CXCR2, **C.** KRT14, and **D.** TNF-α in SGC 7901 cells either treated with anti-PADI4 siRNA or transfected with a PADI4-overexpressing plasmid using real-time PCR. The experiment was repeated three times. To quantify expression in the silenced PADI4 cells, the mRNA expression level of each gene in the AllStar siRNA-treated cells was set to “1”, and the expression level of the target gene in the anti-PADI4 siRNA-treated cells was normalized to the mRNA level of its corresponding gene in the AllStar siRNA-treated cells. To quantify expression in cells overexpressing PADI4, the mRNA expression level of each gene in SGC 7901 cells transfected with empty pcDNA3.1 vector was set to “1”, and the expression level of the target gene in the cells transfected with PADI4-overexpressing plasmid was normalized to its corresponding gene expression in the mock cells. The expression levels are expressed as the mean±standard error of the mean. ** p<0.01 and *** p<0.001.

**Figure 5:**
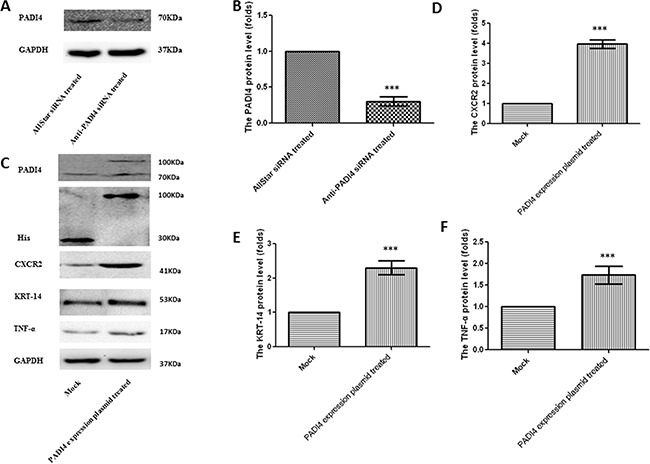
Determination of protein expression levels of PADI4, CXCR2, KRT-14 and TNF-α in SGC 7901 cells either treated with anti-PADI4 siRNA or transfected with a PADI4-overexpressing plasmid using western blot analysis **A.** The protein expression of PADI4 in the siRNA-treated SGC 7901 cells. **B.** PADI4 expression in the siRNA-treated cells was normalized to the GAPDH expression. **C.** The protein expressions of PADI4, His, CXCR2, KRT14 and TNF-α in SGC 7901 cells transfected with a recombinant pcDNA3.1 vector overexpressing PADI4. The expression of CXCR2 **D.**, KRT-14 **E.** and TNF-α **F.** was normalized to the GAPDH expression. The expression levels are expressed as the mean±standard error of the mean. *** p<0.001.

To confirm the universality of these expression changes, we measured the levels of CXCR2, KRT14 and TNF-α in SGC 7901 cells, which are also derived from gastric tumors. The SGC 7901 cells were treated with anti-PADI4 siRNA, and real-time PCR and western blotting detected significantly decreased PADI4 transcription and translation by 10- and 3-fold, respectively, indicating the successful inhibition of PADI4 expression (Figure 4A, Figure 5A-5B). Real-time PCR also detected significantly decreased transcription of CXCR2, KRT14 and TNF-α in SGC 7901 cells treated with anti-PADI4 siRNA relative to the cells treated with AllStar siRNA (Figure 4B-4D).

The recombinant pcDNA3.1 vector with the PADI4 gene insert was then transfected into SGC 7901 cells. The transcription of PADI4 was significantly increased by approximately 60-fold in the SGC 7901 cells transfected with PADI4-expression plasmids compared with the cells transfected with mock plasmids (Figure 4A). Western blotting analysis detected a band at a molecular weight of 100 kDa with an anti-His antibody in extracts of SGC 7901 cells transfected with the recombinant overexpression vector, but only a band at a molecular weight of 30 kDa was detected in extracts of cells transfected with blank pcDNA3.1 vector. Western blotting also detected PADI4 expression at molecular weights of 100 kDa and 70 kDa in the extracts of SGC 7901 cells transfected with the recombinant overexpression vector, whereas only a single PADI4 band at a molecular weight of 70 kDa was detected in the extracts of cells transfected with blank pcDNA3.1 vector (Figure 5C). These observations indicated the successful transfection of the recombinant plasmid and the overexpression of PADI4 in SGC cells (Figure 5C). Real-time PCR analysis detected a significant increase in the expression levels of CXCR2, KRT14 and TNF-α in PADI4-overexpressing SGC 7901 cells. These experiments were repeated three times, and the results are shown in Figure 4B-4D. Western blot analysis detected an approximately 3-fold increase in the protein expression levels of CXCR2, KRT14 and TNF-α in the extracts of SGC 7901 cells overexpressing PADI4 relative to cells transfected with mock plasmid (Figure 5C-5F).

## DISCUSSION

In the present study, we used an Illumina custom-designed SNP microarray to genotype 59 tag SNPs in the PADI4 locus to determine the association of these SNPs to various tumor types. Several SNPs, including rs882537, rs1748034, rs1748032, rs1635566 and rs1635584, showed significant differences with regard to allelic frequency, genotypic frequency or both between the gastric carcinoma samples and the control samples. This genotyping result was verified by the TaqMan assay in an independent cohort of individuals with gastric carcinoma. The analysis showed a significant difference in the allele frequency and the genotype frequency for rs1635566 between the gastric carcinoma samples and the controls. The analysis also detected a significant association of rs882537 and this cancer with regard to the allele frequency. The TaqMan genotyping results are in agreement with the Illumina microarray results. These findings strongly demonstrate that the PADI4 gene has a valid susceptibility to gastric tumor risk. The result encourages us to focus on investigating the tumorigenic role of PADI4 in gastric tumors. In addition, our Illumina SNP microarray assay showed a significant association of rs1635586 with breast cancer and esophageal carcinoma as well as a significant association of rs1635564 with rectal carcinoma. We previously reported that the haplotype GC, which contains rs2501796 and rs2477134 in the PADI4 locus, was significantly associated with an increased risk of esophageal carcinoma [11]. Many studies have also reported that genetic variants in the PADI4 locus are related to rheumatoid arthritis [15, 16].

PADI4 gene is 55810 bp long and has 17 exons. The tag SNP rs1635566/rs59611518 is on the fourteenth intron of the PADI4 gene and 203 bp upstream of the fifteenth exon, and rs882537/rs3829723 is on the second intron and 589 bp upstream of the third exon. All published data regarding the PADI4 SNPs do not indicate a potential bio-function of these two tag SNPs in the intron region. A tag SNP is a representative single nucleotide polymorphism in a region of the genome with high linkage disequilibrium that represents a group of SNPs called a haplotype. It is feasible to genotype a small number of tag SNPs to determine a potential association with phenotypes without genotyping every SNP in a chromosomal region. To investigate the relationship between the pathogenesis of rheumatoid arthritis and the associated haplotypes, Suzuki *et al.* identified four exonic SNPs, three of which are involved in amino acid substitutions: padi4-89, padi4-90, padi4-92 and padi4-104, resulting in Ser55 to Gly, Ala82 to Val and Ala112 to Gly, respectively. The authors found that the mRNA of the susceptible haplotype was more stable than that of the non-susceptible haplotype, suggesting that SNPs in the PADI4-coding region contribute to mRNA stability. Susceptible-haplotype mRNA likely accumulates at higher levels than non-susceptible mRNA, resulting in higher levels of PADI4 protein expression [17]. However, Acta *et al.* found that these three amino acid substitutions only induced conformational changes within the N-terminal domain and did not alter the citrullination activity of PADI4 enzyme. These structural and biochemical analyses suggest that improper protein citrullination in rheumatoid arthritis patients is not caused by defects in citrullination activity but by other reasons such as enhanced PADI4 mRNA stability [18]. To date, we do not know whether the SNPs rs1635566 and rs882537 play a role in enhancing mRNA stability. Normally, SNPs in the middle of an intron do not exert a bio-function to influence either gene expression levels or the activity of the encoded protein product [19]. We will genotype more SNPs in this region, particularly in the exon region, to identify functional SNPs that may affect the expression level or enzymatic activity of PADI4 as well as the related signaling pathway in a future study.

In our previous study, we detected considerably increased transcription and translation of PADI4 in gastric tumor tissues compared with corresponding healthy tissues and stomach leiomyosarcoma tissues using real-time PCR, western blotting and immunohistochemistry [10]. We also detected citrullination of β-tubulin and α-enolase in SGC 7901 cells (which are derived from gastric cancer) using a proteomic method [20]. The increased expression of PADI4 in gastric tumor tissues corresponds to the present finding regarding the significant association of PADI4-encoding DNA variants with gastric carcinoma. To date, citrullination of histones, cytokeratin, antithrombin and fibronectin has been detected and found to be involved in abnormal apoptosis, increased coagulation, and disordered cell proliferation and differentiation, all of which are main features of malignant tumors [6]. These findings suggest that PADI4 may play an important role in the tumorigenesis of gastric tumors.

The tumorigenic pathway of PADI4 was analyzed in the gastric tumor-derived MNK-45 cell line using a series of tumorigenesis-related PCR arrays. RNA interference against PADI4 expression resulted in the significantly altered expression of 10 genes involved in cancer pathways, p53 signaling, signal transduction and tumor metastasis. Real-time PCR and western blotting were then used to verify the PCR array results in MNK-45 cells that either suppress PADI4 expression or overexpress PADI4. Finally, increased mRNA and protein expression levels of CXCR2, KRT14 and TNF-α were detected in the PADI4-overexpressing MNK-45 cells, whereas decreased expression of these three genes was detected in the cells that suppressed PADI4 expression. Moreover, similar results were obtained in SGC 7901 cells, which is another tumor cell line derived from gastric tumors. These results demonstrate that altered PADI4 expression significantly affects CXCR2, KRT14 and TNF-α expression and suggests that PADI4 plays a role in gastric tumorigenesis through the CXCR2, KRT14 and TNF-α pathways. The present study used anti-PADI4 siRNA to transiently knock down PADI4 mRNA expression. We did not establish a MNK-45 cell line that stably inhibited PADI4 expression, which can explain the 4- to 10-fold discrepancy in the decrease in PADI4 mRNA levels between the PCR array and the real-time PCR results. However, we found that PADI4 protein expression levels were 3- to 4-fold lower than those in the control cells without any considerable alterations between the two experiments. This observation suggests that the altered mRNA expression of PADI4 is not always proportional with the altered protein expression, and the discrepancy in the decreased mRNA levels between the two experiments did not considerably affect the downstream gene expression patterns.

It is well known that CXCR2 participates in chronic inflammation, angiogenesis, tumorigenesis and metastasis in several types of carcinomas by binding to its native ligand interleukin-8 (IL-8) [21–31]. The expression level of CXCR2 is higher in gastric cancer tissues than in adjacent noncancerous tissues [32] and is associated with the extent of tumor differentiation, advanced clinical stage, lymph node involvement, and distant metastasis of gastric cancer [33]. KRT14 (keratin 14) is expressed in mitotically active cells of the basal layer along with its partner keratin 5, and the expression of these two proteins is downregulated as cells differentiate. In lymph node metastases of squamous cell carcinoma, tumor cells often express KRT14 in the trabecular nests of the primary carcinoma [34]. KRT14 also plays a role in the maintenance of proliferation potential in cells in the basal layer of stratified epithelia [35]. We previously detected the citrullination of keratin and co-localization of PADI4 with keratin in many tumor tissues [9]. TNF-α is a pleiotropic cytokine that is involved in cell proliferation, differentiation, inflammation and cell death via binding to specific receptors on cell membranes. TNF-α polymorphisms have been found to be associated with gastric carcinogenesis [36–38]. *Helicobacter pylorus,* a definitive carcinogen for stomach cancer, is known to induce the expression of pro-inflammatory cytokines such as TNF-α and interleukin-1 in the stomach [39]. Thus, TNF-α is an endogenous tumor promoter [40]. Park *et al.* reported that the inhibition of PADI4 expression markedly attenuated the TNF-α-induced increase of NF-κB, AP-1 and VCAM-1 (vascular cell adhesion molecule-1) expression in vascular smooth muscular cells [41]. Kim *et al.* also reported that PADI4 siRNA repressed TACE (TNF-α converting enzyme) expression [42]. The current study found that stimulation of PADI4 increased CXCR2, KRT14 and TNF-α expression levels, suggesting that increased expression of PADI4 in gastric tumor tissues, as we previously observed, contributes to tumorigenesis by increasing the expression levels of CXCR2, KRT14 and TNF-α.

p53, a well-known tumor suppressor gene, inhibits malignant transformation through the transcriptional regulation of its target genes, which function to mediate the cell cycle and apoptosis. Studies have indicated that p53 regulates the transcription of target genes by post-translational modification of histones via methylation, acetylation, phosphorylation and ubiquitination [43, 44]. Yao *et al.* and Li *et al.* found that the overexpression of PADI4 may prevent the methylation of histone H3 by converting arginine into citrulline. As a result, the expression of p53 target genes is reduced, which results in the disruption of cellular apoptosis and cell cycle progression [45, 46]. Hagiwara *et al.* and Cuthbert *et al.* also confirmed that the citrullination of histones by PADI4 antagonizes arginine methylation [47, 48]. These findings support the notion that PADI4 overexpression counteracts p53 function. Furthermore, Guo *et al.* found that upregulation of CXCR2 is dependent on the dysfunction of p53 [49]; Cai *et al.* found that p53 represses the promoter activity of the KRT14 promoter and downregulates KRT14 expression [50]; Tang *et al.* found that a transcription factor termed LPS-induced TNF factor (LITAF) significantly induces TNF-α production. Furthermore, p53 specifically downregulates LITAF/TNF-α gene expression [51]. These findings and ours support the possibility that overexpression of PADI4 interrupts the p53 signaling pathway, which in turn leads to increased expression of CXCR2, KRT14 and TNF-α. Recently, we transfected two ovarian cancer cell lines, A2780 (wild-type for p53) and SKOV3 (p53-null), with PADI4-siRNA. The proliferation of both A2780 and SKOV3 cells decreased significantly following anti-PADI4 siRNA treatment. The invasion and migratory ability of the A2780 cells was also significantly decreased in response to PADI4-siRNA treatment, but the SKOV3 cells showed no such decrease. The apoptotic rate of the A2780 cells increased in the presence of PADI4-siRNA, but there was no such increase in SKOV3 cells. These results support the notion that PADI4 plays an important role in the p53 pathway and the regulation of the proliferation, apoptosis, invasion and migration of tumor cells [14].

In summary, the present study demonstrated that PADI4 is a susceptible gene of gastric carcinoma. Moreover, this study found that PADI4 may contribute to cancer development and progression by increasing the expression of CXCR2, KRT14 and TNF-α, which activate pro-inflammatory processes, angiogenesis, cell migration, cell proliferation and cell differentiation in tumor tissues. These findings may be useful in improving our understanding of the tumorigenic process.

## MATERIALS AND METHODS

### Patients and blood sample collection

In the present study, all of the blood samples were collected from patients at Shandong Provincial Qianfoshan Hospital (Jinan, Shandong, China) and PKUCare Luzhong Hospital/Zibo Branch of Shandong Provincial Qianfoshan Hospital (Linzi, Shandong. China). Tumor diagnoses were verified using histology, and pathological categorizations were performed according to the World Health Organization (WHO) classification system. All of the included patients signed informed consent, and this study was approved by the Ethics Committee of Shandong Provincial Qianfoshan Hospital. Blood samples were placed into Monovette tubes containing 3.8% sodium citrate.

### Genomic DNA isolation and SNP selection

Genomic DNA was extracted from whole blood samples using the Omega E-Z 96 Blood DNA kit (Omega, USA) according to the manufacturer's protocol. After extraction, the genomic DNA was diluted to a final concentration of 15-20 ng/μl for the genotyping assays.

Tag single nucleotide polymorphisms (tag SNPs) across the PADI4 locus were determined through a search of the HapMap database. Only SNPs with a minor allele frequency (MAF) greater than 5% and a pair-wise r^2^ ≥ 0.8 were considered. The Illumina Assay Design Tool filtered out SNPs that were not suitable for the Illumina platform, including insertions/deletions, tri- and tetra-allelic SNPs, and SNPs that were not uniquely localized. Fifty-nine SNPs in the PADI4 locus with a design score of 1 were selected; these SNPs spanned 55,600 bases of chromosome 1p36.13. These SNP sites and locations are described in Supplementary Table S2.

### Genotyping assay using an Illumina 384-SNP VeraCode microarray

We performed a genotyping assay using a custom-designed Illumina 384-SNP VeraCode microarray (Illumina). Peripheral blood samples were collected from patients with breast cancer (n=281, 281 females, aged 26-80 years, mean age 49.3 years), cervical carcinoma (n=202, 202 females, aged 29-61 years, mean age 54.4 years), esophageal carcinoma (n=157, 30 females, aged 39-86 years, mean age 60.8 years), gastric carcinoma (n=221, 64 females, aged 31-80 years, mean age 58.2 years), liver cancer (n=202, 30 females, aged 30-79 years, mean age 55.9 years), ovarian cancer (n=197, 197 females, aged 19-76 years, mean age 52.9 years) or rectal carcinoma (n=101, 36 females, aged 30-75 years, mean age 55.6 years). A total of 384 healthy individuals (125 females, aged 24-58 years) with a mean age of 40.2 years served as control blood donors. Genotyping was conducted with a BeadXpress Reader using the Illumina VeraCode Golden Gate Assay kit. A total of 500 ng of sample DNA was used per assay. Genotype clustering and calling were performed using BeadStudio software (Illumina). This work was completed at the Beijing Institute of Genomics of the Chinese Academy of Sciences.

### Taqman genotyping assay

To verify the Illumina genotyping results, tag SNPs of rs41265997, rs1635566, rs882537, rs1635586 and rs1748034 were selected for the genotyping of cohorts of patients with gastric cancer (n=190, 64 women, mean age=57.7) and healthy controls (n=288, 72 women, mean age=40). These cohorts are different from cohorts examined by the above mentioned Illumina SNP microarray assay. The genomic DNA was extracted as described above. The genomic DNA was diluted to a final concentration of 15-20 ng/μl for the genotyping assays. The assays were run in a ViiA 7 DX real-time PCR machine (Life Technologies, USA) and evaluated according to the manufacturer's instructions. The reactions were performed in a total volume of 10 μl using the following amplification protocol: denaturation at 95°C for 10 minutes, followed by 50 cycles of denaturation at 95°C for 15 seconds and then annealing and extension at 60°C for 1 minute. The genotype of each sample was determined by measurement of allele-specific fluorescence using Taqman Genotyper software V1.2 (Life Technologies). Duplicate samples and negative controls were included to verify the accuracy of the genotyping results.

The genotyping quality was examined by a detailed quality control procedure that consisted of a > 95% successful call rate, duplicate calling of the genotypes, internal positive control samples and HWE tests. The SNPs were analyzed for association by a comparison of the MAF between the cases and controls. Dominant and recessive models were considered with respect to the minor allele. The association of the SNPs with diseases was evaluated by odds ratios (OR) with 95% confidence intervals (CI). Fisher's exact test was used for comparisons between categorical variables. P values less than 0.05 were considered statistically significant. Genotypic association was assessed with Plink v1.07 (http://pngu.mgh.harvard.edu/purcell/plink/) and SHEsis (http://analysis.bio-x.cn/myAnalysis.php) software [52, 53]. Bonferroni single-step correction was performed by Plink v1.07.

### Cell culture and transfection of anti-PADI4 siRNA or the PADI4-overexpressing plasmid into MNK-45 cells and SGC7901 cells

The gastric cancer cell line MNK-45 was cultured in RPMI 1640 medium supplemented with 10% fetal calf serum, 50 U/mL penicillin and 50 μg/mL streptomycin in an atmosphere of 5% CO_2_ at 37°C. An siRNA oligonucleotide that targets the PADI4 gene (target sequence: 5′CAGCGTAGTCTTGGGTCCCAA 3′) was designed and synthesized by Qiagen (Germany). The cultured tumor cells were transfected with siRNA at a concentration of 20 nM using the HiPerFect transfection reagent (Qiagen) according to the manufacturer's protocol. The cells were harvested for analysis 48 h after transfection. Parallel experiments were conducted with Mm/Hs-MAPK1 siRNA (AATGCTGACTCCAAAGCTCTG) and AllStar siRNA, both of which were provided with the kit, and these sequences were used as positive and negative controls, respectively. The control RNAs were used at the same concentrations as the PADI4 siRNA. The inhibition of PADI4 expression in these cell lines was verified by real-time PCR and western blotting.

The full coding sequence of PADI4 is 1,992 bp long and codes for 663 amino acid residues. The full coding sequence was amplified from cDNA that was reverse-transcribed from total RNA of the gastric tumor tissues using the specific primers PADI4-EcoRI-Fex (TACTCAGAATTCATGGCCCAGGGGACATTG) and PADI4-AscI-Rex (TACTCAGGCGCGCCGAGGGCACCATGTTCCA). The PCR product was then sequenced and inserted into the pcDNA 3.1(+) expression vector (Invitrogen), which is a 5.4 kb vector derived from pcDNA3, is designed for high-level, constitutive expression of recombinant protein in a variety of mammalian cell lines, and contains a cleavable N-terminal 6xHis tag. The recombinant plasmid was purified with a Plasmid Mini kit based on the manufacturer's protocol (Sangon Biotech, China). The MNK-45 cells were cultured until they reached approximately 70% confluence, after which the RPMI 1640 medium was replaced with medium containing no fetal bovine serum, and the cells then remained in culture for 1 h at 37°C. Afterward, 2 μg of plasmid DNA was diluted in 125 μl of 1640 medium (without antibiotics and serum), vortexed, and then incubated for 10 min at room temperature. Concurrently, 8 μl of DNAfectin Transfection Reagent (Tiangen, China) was diluted in 125 μl of 1640 medium (without antibiotics or serum), briefly mixed and vortexed, and incubated at room temperature 10 min. The diluted DNA mixture was combined with the diluted DNAfectin Transfection Reagent, mixed gently, and then incubated for 20 min. The DNA-lipid mixture was then added dropwise into the well and incubated with 1640 medium (without antibiotics and serum). After incubation for 12 h, the 1640 medium (without antibiotics or serum) was discarded, and the cells were cultured for at least 24 h in 1640 medium with 10% FBS. The expression of recombinant PADI4 protein was detected using Western blot analysis as described below.

SGC 7901 cells that originated from gastric tumor were transfected with either anti-PADI4 siRNA or the PADI4 overexpression vector as described above.

### PCR array analysis

Total RNA was isolated from the anti-PADI4 siRNA-treated MNK-45 cells with TRIzol solution (Invitrogen, USA) according to the manufacturer's protocol. The PCR arrays are sets of optimized real-time PCR primer assays in 96-well plates that are used to monitor the expression of genes related to a disease state or pathway. In this study, the Cancer Pathway Finder, p53 Signaling Pathway, Signal Transduction and Tumor Metastasis PCR arrays (Qiagen) were used to identify the pathogenic pathways of PADI4 in the genesis of gastric cancer. The PCR array analysis was conducted using a ViiA7 DX system (Life Science) according to the manufacturer's protocol. The procedure begins with the conversion of experimental RNA samples to single-stranded cDNA using the RT^2^ First Strand Kit. Next, the cDNA is mixed with an appropriate amount of RT^2^ SYBR Green MasterMix. This mixture is then distributed into the wells of the RT^2^ Profiler PCR Array. After the PCR reaction is executed, the relative expression level is determined using data from the real-time cycler and the ΔΔCT method. The raw array data were processed and analyzed by the PCR Array Data Analysis System at http://sabiosciences.com/pcrarraydataanalysis.php. Fold changes were calculated and expressed as log-normalized ratios of the anti-PADI4 siRNA-treated cells/AllStar siRNA-treated cells. Based on the manufacturer's guidelines, genes with at least a 4-fold change in expression were considered to be biologically significant in the study.

### Real-time PCR

Total RNA was extracted from either the anti-PADI4 siRNA-treated MNK-45 cells or MNK-45 cells transfected with the PADI4 overexpression pcDNA3.1 plasmid. The total RNA was reverse-transcribed in a final volume of 10 μl using an RNA PCR kit (TaKaRa, Japan). Real-time PCR reactions were performed in a ViiA7 DX Instrument (Life Technologies, USA) according to the manufacturer's protocol. The PCR reactions were performed in a total volume of 10 μl, which contained 1 μl of cDNA, 5 μl of SYBR Green Real-time PCR Master Mix (ToYoBo, Japan) and 1 μl of each primer. The PCR amplification cycles were performed under the following conditions: 10 s at 95°C, and 45 cycles of 15 s at 95°C and 60 s at 60°C. For each sample, two reactions were performed simultaneously: the first determined the mRNA level of the target gene, and the second determined the mRNA level of β-actin. The PCR products were confirmed by a melt curve analysis. The relative mRNA expression was calculated using the comparative threshold cycle (Ct) method. The relative target gene expression levels were normalized to the expression level of β–actin mRNA. The forward and reverse primer sequences for the gene amplification were as follows: PADI4, 5′GGGGTGGTCGTGGATATTGC3′ and 5′CCCGGTGAGGTAGAGTAGAGC3′; β-actin, 5′TGGCACCCAGCACAATGAA3′and 5′CTAAGTCA TAGTCCGCCTAGAAGCA3′; G6PD, 5′CCAACAGGA TCTTCGGC3′ and 5′ACACAGCATCTGCAGTAG3′; CA9, 5′GCCTTTCTGGAGGAGGG3′ and 5′AGATAT GTCCAGTCCTGGG3′; IGFBP3, 5′CTACGAGTCTCA GAGCACAGATA3′ and 5′ATTCAGTGTGTCTTCCAT TTCTCTA3′; KRT14, 5′TGTGGAGATGGACGCTG3′ and 5′ATTCCTCGGCATCCTTG3′; CCL5, 5′GCCCAC ATCAAGGAGTAT3′ and 5′CAGGAAATCCTGCCAG AC3′; MMP7, 5′CCAAACTCAAGGAGATGCAA3′ and 5′AACATCTGGCACTCCAC3′; TNF-α, 5′CCAGG GACCTCTCTCTAATC3′ and 5′CTTGAGGGTTTGCTA CAACA3′; MMP2, 5′ACCGCGACAAGAAGTAT3′ and 5′ATTTGTTGCCCAGGAAAG3′; and CXCR2, 5′AG GTGAATGGCTGGATTT and 5′CACTGATGCAGGCC AGTA3′.

The gene expression pattern in the SGC 7901 cells either treated with anti-PADI4 siRNA or transfected with pcDNA3.1 plasmid overexpressing PADI4 was determined using the same protocol.

### Western blot analysis

Cultured MNK-45 and SGC 7901 cells were respectively homogenized in cell lysis solution (Sigma) and centrifuged at 12,000 xg for 30 min at 4°C. Supernatants were collected after centrifugation, and protein concentrations were determined using a BCA Protein Assay kit (Pierce). In total, 30 μg of protein was loaded and separated by sodium dodecyl sulfate-polyacrylamide gel electrophoresis (SDS-PAGE), followed by transfer onto a polyvinylidene membrane and treatment with a rabbit anti-human PADI4 antibody (Sigma). This antibody targets amino acids 91-107 of human PADI4. Western blotting performed by manufacturer demonstrated that this antibody has no cross-reactivity among other PAD family members. The polyvinylidene membranes were subsequently rinsed with a wash solution and incubated with sheep anti-rabbit IgG conjugated to peroxidase (Beyotime Biotech) for 1 h. Following another wash step, the signal was detected using an enhanced chemiluminescence (ECL) plus kit (Beyotime Biotech). Another membrane was prepared using the same protocol and probed with an anti-GADPH antibody (Santa Cruz) to normalize sample loading.

The expression levels of CXCR2, KRT14, TNF-α and the His tag in the cultured cells were examined by the same western protocol described above. Commercial antibodies against these proteins were obtained from CST, Sigma, Abcam and Abcam, respectively.

### Statistical analysis

Data were analyzed by a two-tailed Student's t-test. Differences were considered to be statistically significant at p<0.05. To verify the results, each experiment was performed with three samples in triplicate.

## SUPPLEMENTARY FIGURES AND TABLES







## References

[1] Arita K, Hashimoto H, Shimizu T, Nakashima K, Yamada M, Sato M (2004). Structural basis for Ca(2+)-induced activation of human PAD4. Nat Struct Mol Biol.

[2] Chavanas S, Méchin MC, Nachat R, Adoue V, Coudane F, Serre G, Simon M (2006). Peptidylarginine deiminases and deimination in biology and pathology: relevance to skin homeostasis. J Dermatol Sci.

[3] Pritzker LB, Nguyen TA, Moscarello MA (1999). The developmental expression and activity of peptidyl arginine deiminase in the mouse. Neurosci Lett.

[4] Mohanan S, Cherrington BD, Horibata S, McElwee JL, Thompson PR, Coonrod SA (2012). Potential role of peptidyl arginine deiminase enzymes and protein citrullination in cancer pathogenesis. Biochem Res Int.

[5] Méchin MC, Coudane F, Adoue V, Arnaud J, Duplan H, Charveron M, Schmitt AM, Takahara H, Serre G, Simon M (2010). Deimination is regulated at multiple levels including auto-deimination of peptidyl arginine deiminases. Cell Mol Life Sci.

[6] Chang X, Fang K (2010). PADI4 and Tumorigenesis. Cancer Cell International.

[7] Kolodziej S, Kuvardina ON, Oellerich T, Herglotz J, Backert I, Kohrs N, Buscató El, Wittmann SK, Salinas-Riester G, Bonig H, Karas M, Serve H, Proschak E, Lausen J (2014). PADI4 acts as a coactivator of Tal1 by counteracting repressive histone arginine methylation. Nat Commun.

[8] Tanikawa C, Espinosa M, Suzuki A, Masuda K, Yamamoto K, Tsuchiya E, Ueda K, Daigo Y, Nakamura Y, Matsuda K (2012). Regulation of histone modification and chromatin structure by the p53-PADI4 pathway. Nat Commun.

[9] Chang X, Han J (2006). The expression of PAD4 in various tumors. Molecular Carcinogenesis.

[10] Chang X, Han J, Pang L, Zhao Y, Yang Y, Shen Z (2009). PADI4 Has Increased Expression In Blood and Tissues of Malignant Tumors. BMC cancer.

[11] Chang X, Hou X, Pan J, Fang K, Wang L, Han J (2011). Investigating the Pathogenic Role of PADI4 in Oesophageal Cancer. International Journal of Biomedical Science.

[12] Ordóñez A, Yélamos J, Pedersen S, Miñano A, Conesa-Zamora P, Kristensen SR, Stender MT, Thorlacius-Ussing O, Martínez-Martínez I, Vicente V, Corral J (2010). Increased levels of citrullinatedantithrombin in plasma of patients with rheumatoid arthritis and colorectal adenocarcinoma determined by a newly developed ELISA using a specific monoclonal antibody. ThrombHaemost.

[13] Wang L, Chang X, Yuan G, Zhao Y, Wang P (2010). Expression of peptidylarginine deiminase type 4 in ovarian tumors. Int J Biol Sci.

[14] Cui Y, Yan L, Zhou J, Zhao S, Zheng Y, Sun B, Lv H, Rong F, Chang X (2016). The role of peptidylarginine deiminase 4 in ovarian cancer cell tumorigenesis and invasion. Tumour Biol.

[15] Suzuki A, Yamamoto K (2015). From genetics to functional insights into rheumatoid arthritis. ClinExpRheumatol.

[16] Too CL, Murad S, Dhaliwal JS, Larsson P, Jiang X, Ding B, Alfredsson L, Klareskog L, Padyukov L (2012). Polymorphisms in peptidylarginine deiminase associate with rheumatoid arthritis in diverse Asian populations: evidence from MyEIRA study and meta-analysis. Arthritis Res Ther.

[17] Suzuki A, Yamada R, Chang X, Tokuhiro S, Sawada T, Suzuki M, Nagasaki M, Nakayama-Hamada M, Kawaida R, Ono M, Ohtsuki M, Furukawa H, Yoshino S, Yukioka M, Tohma S, Matsubara T, Wakitani S, Teshima R, Nishioka Y, Sekine A, Iida A, Takahashi A, Tsunoda T, Nakamura Y, Yamamoto K (2003). Functional haplotypes of PADI4, encoding citrullinating enzyme peptidylarginine deiminase 4, are associated with rheumatoid arthritis. Nat Genet.

[18] Horikoshi N, Tachiwana H, Saito K, Osakabe A, Sato M, Yamada M, Akashi S, Nishimura Y, Kagawa W, Kurumizaka H (2011). Structural and biochemical analyses of the human PAD4 variant encoded by a functional haplotype gene. ActaCrystallogr D Biol Crystallogr.

[19] Samuels DC, Han L, Li J, Quanghu S, Clark TA, Shyr Y, Guo Y (2013). Finding the lost treasures in exome sequencing data. Trends Genet.

[20] Jiang Z, Cui Y, Wang L, Zhao Y, Yan S, Chang X (2013). Investigating citrullinated proteins in tumor cell lines. World J Surg Oncol.

[21] Gabellini C, Trisciuoglio D, Desideri M, Candiloro A, Ragazzoni Y, Orlandi A, Zupi G, Del Bufalo D (2009). Functional activity of CXCL8 receptors, CXCR1 and CXCR2, on human malignant melanoma progression. Eur J Cancer.

[22] Yang J, Su Y (2009). The good and the bad of chemokines/chemokine receptors in melanoma. Pigment Cell Melanoma Res.

[23] Baier PK, Wolff-Vorbeck G, Eggstein S, Baumgartner U, Hopt UT (2005). Cytokine expression in colon carcinoma. Anticancer Res.

[24] Ohri CM, Shikotra A, Green RH, Waller DA, Bradding P (2010). Chemokine receptor expression in tumour islets and stroma in non-small cell lung cancer. BMC Cancer.

[25] Liu Z, Yang L, Xu J, Zhang X, Wang B (2011). Enhanced expression and clinical significance of chemokine receptor CXCR2 in hepatocellular carcinoma. J SurgRes.

[26] Mestas J, Burdick MD, Reckamp K, Pantuck A, Figlin RA, Strieter RM (2005). The role of CXCR2/CXCR2 ligand biological axis in renal cell carcinoma. J Immunol.

[27] Wang B, Hendricks DT, Wamunyokoli F, Parker MI (2006). A growth-related oncogene/CXC chemokine receptor 2 autocrine loop contributes to cellular proliferation in esophageal cancer. Cancer Res.

[28] Matsuo Y, Ochi N, Sawai H, Yasuda A, Takahashi H, Funahashi H, Takeyama H, Tong Z, Guha S (2009). CXCL8/IL-8 and CXCL12/SDF-1alpha co-operatively promote invasiveness and angiogenesis in pancreatic cancer. Int J Cancer.

[29] Yang G, Rosen DG, Liu G, Yang F, Guo X, Xiao X, Xue F, Mercado-Uribe I, Huang J, Lin SH, Mills GB, Liu J (2010). CXCR2 promotes ovarian cancer growth through dysregulated cell cycle, diminished apoptosis, and enhanced angiogenesis. Clin Can Res.

[30] Han L, Jiang B, Wu H, Wang X, Tang X, Huang J, Zhu J (2012). High expression of CXCR2 is associated with tumorigenesis, progression, and prognosis of laryngeal squamous cell carcinoma. Med Oncol.

[31] Li A, Cheng XJ, Moro A, Singh RK, Hines OJ, Eibl G (2011). CXCR2-Dependent endothelial progenitor cell mobilization in pancreatic cancer growth. Transl Oncol.

[32] Cheng WL, Wang CS, Huang YH, Tsai MM, Liang Y, Lin KH (2011). Overexpression of CXCL1 and its receptor CXCR2 promote tumor invasion in gastric cancer. Ann Oncol.

[33] Li Z, Wang Y, Dong S, Ge C, Xiao Y, Li R, Ma X, Xue Y, Zhang Q, Lv J, Tan Q, Zhu Z, Song X, Tan J (2014). Association of CXCR1 and 2 expressions with gastric cancer metastasis in ex vivo and tumor cell invasion in vitro. Cytokine.

[34] Tsubokawa F, Nishisaka T, Takeshima Y, Inai K (2002). Heterogeneity of expression of cytokeratin subtypes in squamous cell carcinoma of the lung: with special reference to CK14 overexpression in cancer of high-proliferative and lymphogenous metastatic potential. Pathol Int.

[35] Alam H, Sehgal L, Kundu ST, Dalal SN, Vaidya MM (2011). Novel function of keratins 5 and 14 in proliferation and differentiation of stratified epithelial cells. MolBiol Cell.

[36] Yang JP, Hyun MH, Yoon JM, Park MJ, Kim D, Park S (2014). Association between TNF-α-308 G/A gene polymorphism and gastric cancer risk: a systematic review and meta-analysis. Cytokine.

[37] Rokkas T, Sechopoulos P, Pistiolas D, Kothonas F, Margantinis G, Koukoulis G (2014). Population differences concerning TNF-α gene polymorphisms in gastric carcinogenesis based on meta-analysis. Ann Gastroenterol.

[38] Guo XF, Wang J, Yu SJ, Song J, Ji MY, Cao Z, Zhang JX, Wang J, Dong WG (2013). TNF-α-308 polymorphism and risk of digestive system cancers: a meta-analysis. World J Gastroenterol.

[39] Suganuma M, Kuzuhara T, Yamaguchi K, Fujiki H (2006). Carcinogenic role of tumor necrosis factor-alpha inducing protein of Helicobacter pylori in human stomach. J BiochemMol Biol.

[40] Balkwill F (2006). TNF-alpha in promotion and progression of cancer. Cancer Metastasis Rev.

[41] Park B, Yim JH, Lee HK, Kim BO, Pyo S (2015). Ramalin inhibits VCAM-1 expression and adhesion of monocyte to vascular smooth muscle cells through MAPK and PADI4-dependent NF-kB and AP-1 pathways. Biosci Biotechnol Biochem.

[42] Kim EH, Kim IH, Ha JA, Choi KT, Pyo S, Rhee DK (2013). Antistress effect of red ginseng in brain cells is mediated by TACE repression via PADI4. J Ginseng Res.

[43] Qian H, Wang T, Naumovski L, Lopez CD, Brachmann RK (2002). Groups of p53 arget genes involved in specific p53 downstream effects cluster intodifferent classes of DNA binding sites. Oncogene.

[44] Kaneshiro K, Tsutsumi S, Tsuji S, Shirahige K, Aburatani H (2007). An integratedmap of p53-binding sites and histone modification in the human ENCODE regions. Genomics.

[45] Yao H, Li P, Venters BJ, Zheng S, Thompson PR, Pugh BF, Wang Y (2008). Histone Arg modifications and p53 regulate the expression of OKL38, amediator of apoptosis. J Biol Chem.

[46] Li P, Yao H, Zhang Z, Li M, Luo Y, Thompson PR, Gilmour DS, Wang Y (2008). Regulation of p53 target gene expression by peptidylargininedeiminase. Mol Cell Biol.

[47] Hagiwara T, Hidaka Y, Yamada M (2005). Deimination of Histone H2A and H4 at Arginine 3 in HL-60 Granulocytes. Biochemistry.

[48] Cuthbert GL, Daujat S, Snowden AW, Erdjument-Bromage H, Hagiwara T, Yamada M, Schneider R, Gregory PD, Tempst P, Bannister AJ, Kouzarides T (2004). Histone deimination antagonizes arginine methylation. Cell.

[49] Guo H, Liu Z, Xu B, Hu H, Wei Z, Liu Q, Zhang X, Ding X, Wang Y, Zhao M, Gong Y, Shao C (2013). Chemokine receptor CXCR2 is transactivated by p53 and induces p38-mediated cellular senescence in response to DNA damage. Aging Cell.

[50] Cai BH, Hsu PC, Hsin IL, Chao CF, Lu MH, Lin HC, Chiou SH, Tao PL, Chen JY (2012). p53 acts as a co-repressor to regulate keratin 14 expression during epidermal cell differentiation. PLoS One.

[51] Tang X, O'Reilly A, Asano M, Merrill JC, Yokoyama KK, Amar S (2011). p53 peptide prevents LITAF-induced TNF-alpha-mediated mouse lung lesions and endotoxic shock. CurrMol Med.

[52] The International HapMap Consortium (2004). Integrating ethics and science in the International HapMap Project. Nature Reviews Genetics.

[53] Li Z, Zhang Z, He Z, Tang W, Li T, Zeng Z, He L, Shi Y (2009). Apartition-ligation-combination-subdivision EM algorithm for haplotype inference with multiallelic markers: update of the SHEsis. Cell Res.

