# Supplementary material for: PADI4 has genetic susceptibility to gastric carcinoma and upregulates CXCR2, KRT14 and TNF-α expression levels
Source: Oncotarget. 2016 Aug 19;7(38):62159–76. doi: 10.18632/oncotarget.11398 (PMC5308718; doi:10.18632/oncotarget.11398)
Supplement: Supplementary file 1 [file oncotarget-07-62159-s001.pdf]

PADI4 has genetic susceptibility to gastric carcinoma and upregulates CXCR2, KRT14 and TNF-α expression levels

SUPPLEMENTARY FIGURES AND TABLES

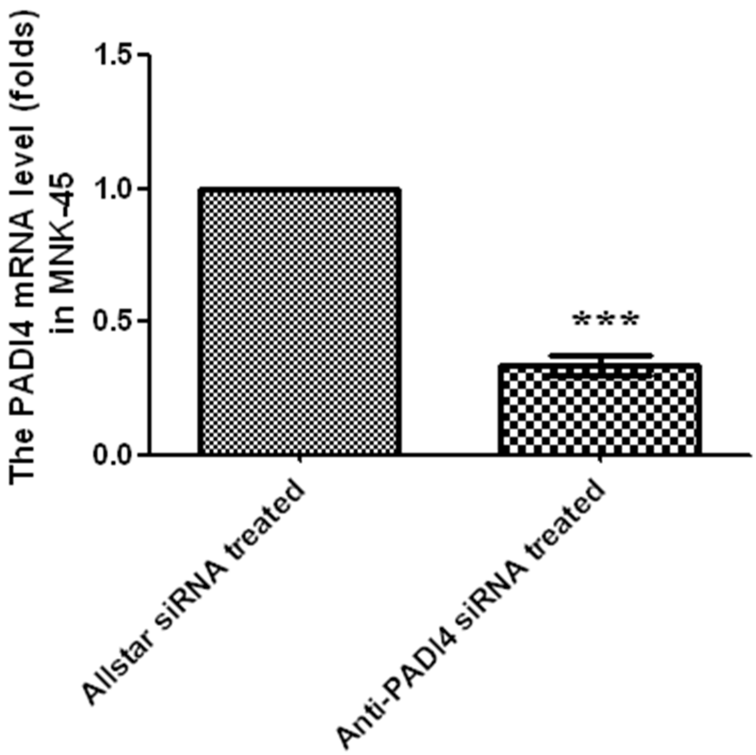

Supplementary Figure S1: Determination of the mRNA expression level of PADI4 in MNK-45 cells treated with anti-PADI4 siRNA. The cells transfected with AllStar siRNA were used as a control. The mRNA level of PADI4 was examined using real-time PCR analysis. The expression level of PADI4 in AllStar siRNA-treated cells was set to "1", and the expression level of PADI4 in the anti-PADI4 siRNA-treated cells was normalized to the level in the AllStar siRNA-treated cells. The expression levels are expressed as the mean±standard error of the mean. *** $p<0.001$.

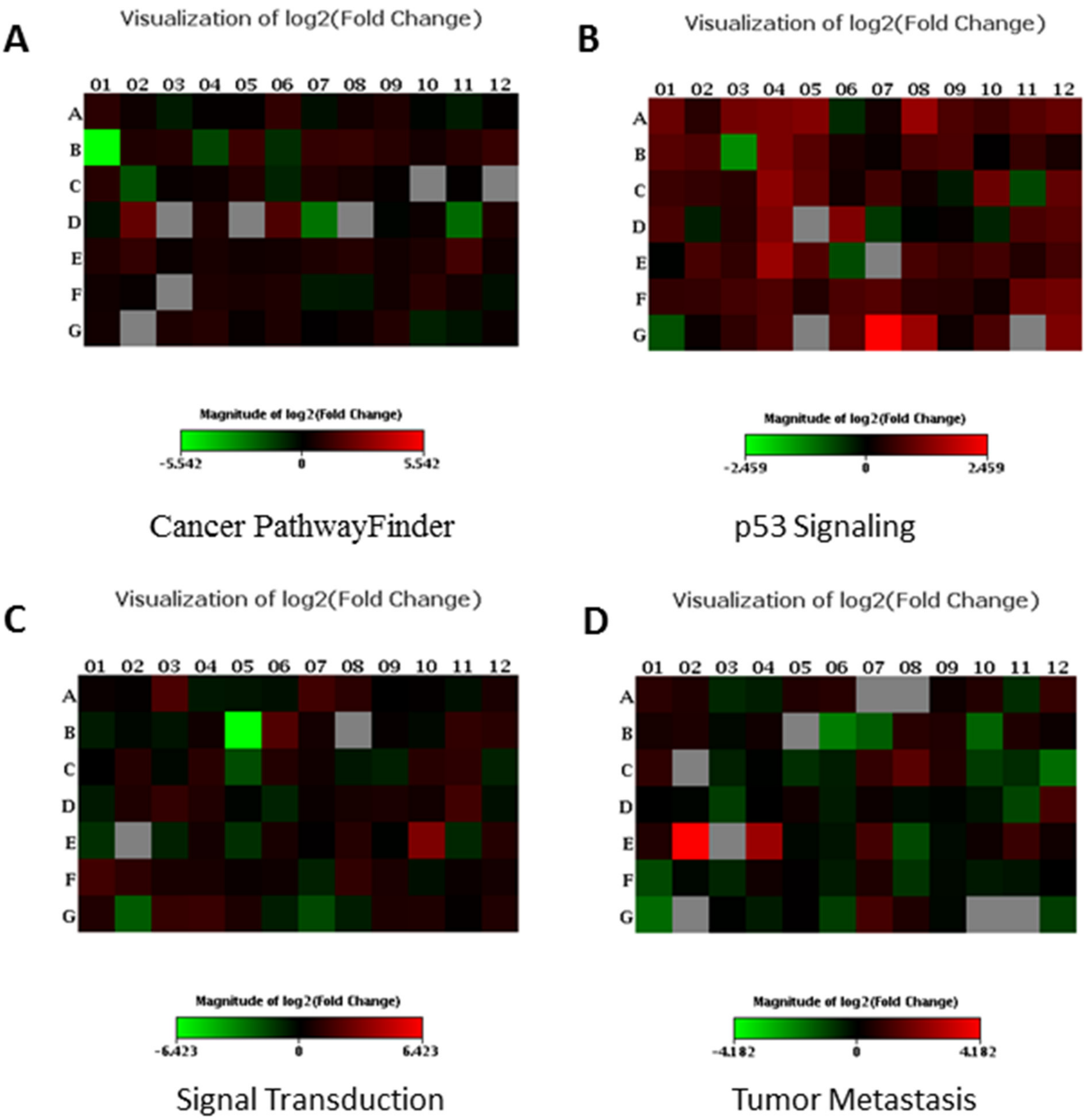

Supplementary Figure S2: Determination of the pathogenic signaling pathway of PADI4 using a series of PCR arrays. MNK-45 cells were treated with anti-PADI4 siRNA. **A.** Cancer Pathwayfinder, **B.** p53 Signaling, **C.** Signal Transduction and **D.** Tumor Metastasis PCR arrays were used to detect altered expression of tumor-related genes in the treated cells. Fold changes were calculated and expressed as the log-normalized ratios of the siRNA-treated cells/controls. The PCR array results are shown as heat maps. Genes that showed at least a 4-fold change in expression were considered to be biologically significant.

Supplementary Table S1: Determination of the pathogenic signaling pathway of PADI4 using a series of PCR arrays. MNK-45 cells were treated with anti-PADI2 siRNA. **A.** Cancer Pathwayfinder, **B.** p53 Signaling, **C.** Signal Transduction and **D.** Tumor Metastasis PCR arrays were used to detect altered expression of tumor-related genes in the treated cells. Fold changes were calculated and expressed as the log-normalized ratios of the siRNA-treated cells/controls. The PCR array results are shown in the tables. Genes that showed at least a 4-fold change in expression were considered to be biologically significant.

See Supplementary File 1

Supplementary Table S2: SNP information.

See Supplementary File 2
