# Supplementary material for: PADI4 has genetic susceptibility to gastric carcinoma and upregulates CXCR2, KRT14 and TNF-α expression levels
Source: Oncotarget. 2016 Aug 19;7(38):62159–76. doi: 10.18632/oncotarget.11398 (PMC5308718; doi:10.18632/oncotarget.11398)
Supplement: Supplementary file 2 [file oncotarget-07-62159-s002.docx]

**Analysis results of Cancer PathwayFinder PCR array**

|  | Layout | 1 | 2 | 3 | 4 | 5 | 6 | 7 | 8 | 9 | 10 | 11 | 12 |
| --- | --- | --- | --- | --- | --- | --- | --- | --- | --- | --- | --- | --- | --- |
| A | Genes | ACLY | ACSL4 | ADM | ANGPT1 | ANGPT2 | APAF1 | ARNT | ATP5A1 | AURKA | BCL2L11 | BIRC3 | BMI1 |
|  | folds | 1.91 | 1.25 | -1.47 | 1.06 | 1.05 | 2.07 | -1.25 | 1.3 | 1.56 | -1.05 | -1.48 | 1.06 |
|  | comments | OKAY | OKAY | OKAY | B | B | OKAY | OKAY | OKAY | OKAY | OKAY | OKAY | OKAY |
| B | Genes | CA9 | CASP2 | CASP7 | CASP9 | CCL2 | CCND2 | CCND3 | CDC20 | CDH2 | CFLAR | COX5A | CPT2 |
|  | folds | -46.59 | 1.57 | 1.67 | -2.8 | 2.39 | -1.95 | 2.11 | 2.16 | 1.75 | 1.37 | 1.74 | 2.28 |
|  | comments | A | OKAY | OKAY | OKAY | A | B | OKAY | OKAY | OKAY | OKAY | OKAY | OKAY |
| C | Genes | DDB2 | DDIT3 | DKC1 | DSP | E2F4 | EPO | ERCC3 | ERCC5 | ETS2 | FASLG | FGF2 | FLT1 |
|  | folds | 1.75 | -3.2 | 1.12 | 1.21 | 1.66 | -1.66 | 1.6 | 1.38 | 1.1 | 1.1 | 1.06 | 1.1 |
|  | comments | OKAY | OKAY | OKAY | OKAY | OKAY | B | OKAY | OKAY | OKAY | C | OKAY | C |
| D | Genes | FOXC2 | G6PD | GADD45G | GPD2 | GSC | HMOX1 | IGFBP3 | IGFBP5 | IGFBP7 | KDR | KRT14 | LDHA |
|  | folds | -1.26 | 4.36 | 1.1 | 1.59 | 1.1 | 3.11 | -5.62 | 1.1 | -1.07 | 1.21 | -4.97 | 1.55 |
|  | comments | OKAY | OKAY | C | OKAY | C | OKAY | OKAY | C | OKAY | B | B | OKAY |
| E | Genes | LIG4 | LPL | MAP2K1 | MAP2K3 | MAPK14 | MCM2 | MKI67 | NOL3 | OCLN | PFKL | PGF | PINX1 |
|  | folds | 1.59 | 2.13 | 1.17 | 1.43 | 1.29 | 1.31 | 1.51 | 1.7 | 1.33 | 1.52 | 2.74 | 1.28 |
|  | comments | OKAY | B | OKAY | OKAY | OKAY | OKAY | OKAY | OKAY | OKAY | OKAY | OKAY | OKAY |
| F | Genes | POLB | PPP1R15A | SERPINB2 | SERPINF1 | SKP2 | SLC2A1 | SNAI1 | SNAI2 | SNAI3 | SOD1 | SOX10 | STMN1 |
|  | folds | 1.28 | 1.12 | 1.1 | 1.53 | 1.42 | 1.55 | -1.42 | -1.38 | 1.3 | 1.82 | 1.35 | -1.24 |
|  | comments | OKAY | OKAY | C | OKAY | OKAY | OKAY | OKAY | OKAY | OKAY | OKAY | B | OKAY |
| G | Genes | TBX2 | TEK | TEP1 | TERF1 | TERF2IP | TINF2 | TNKS | TNKS2 | UQCRFS1 | VEGFC | WEE1 | XIAP |
|  | folds | 1.31 | 1.1 | 1.54 | 1.71 | 1.14 | 1.53 | 1.04 | 1.19 | 1.67 | -1.6 | -1.32 | 1.16 |
|  | comments | B | C | OKAY | OKAY | OKAY | OKAY | OKAY | OKAY | OKAY | OKAY | OKAY | OKAY |

**Analysis results of p53 Signaling PCR array**

|  | Layout | 1 | 2 | 3 | 4 | 5 | 6 | 7 | 8 | 9 | 10 | 11 | 12 |
| --- | --- | --- | --- | --- | --- | --- | --- | --- | --- | --- | --- | --- | --- |
| A | Genes | APAF1 | ATM | ATR | BAI1 | BAX | BBC3 | BCL2 | BCL2A1 | BID | BIRC5 | BRCA1 | BRCA2 |
|  | folds | 2.01 | 1.3 | 2.14 | 2.25 | 2.43 | -1.31 | 1.15 | 2.77 | 1.67 | 1.46 | 1.76 | 1.96 |
|  | comments | OKAY | OKAY | OKAY | B | OKAY | OKAY | OKAY | B | OKAY | OKAY | OKAY | OKAY |
| B | Genes | BTG2 | CASP2 | CASP9 | CCNB1 | CCNE1 | CCNG1 | CCNH | CDC25A | CDC25C | CDK1 | CDK4 | CDKN1A |
|  | folds | 1.79 | 1.65 | -2.55 | 2.25 | 1.78 | 1.17 | 1.07 | 1.55 | 1.64 | 1.01 | 1.41 | 1.18 |
|  | comments | OKAY | OKAY | OKAY | OKAY | OKAY | OKAY | OKAY | OKAY | OKAY | OKAY | OKAY | OKAY |
| C | Genes | CDKN2A | CHEK1 | CHEK2 | CRADD | DNMT1 | E2F1 | E2F3 | EGFR | EGR1 | EI24 | ESR1 | FADD |
|  | folds | 1.47 | 1.4 | 1.31 | 2.58 | 1.86 | 1.13 | 1.53 | 1.13 | -1.18 | 2.04 | -1.6 | 1.89 |
|  | comments | OKAY | OKAY | OKAY | OKAY | OKAY | OKAY | OKAY | OKAY | OKAY | OKAY | B | OKAY |
| D | Genes | FAS | FASLG | FOXO3 | GADD45A | GML | HDAC1 | HK2 | IGF1R | IL6 | JUN | KAT2B | KRAS |
|  | folds | 1.61 | -1.21 | 1.29 | 2.34 | -1.31 | 2.32 | -1.44 | -1.01 | 1.05 | -1.26 | 1.62 | 1.76 |
|  | comments | OKAY | B | OKAY | OKAY | C | OKAY | OKAY | OKAY | OKAY | OKAY | OKAY | OKAY |
| E | Genes | MCL1 | MDM2 | MDM4 | MLH1 | MSH2 | MYC | MYOD1 | NF1 | NFKB1 | PCNA | PIDD | PPM1D |
|  | folds | -1.01 | 1.6 | 1.37 | 2.8 | 1.71 | -1.64 | -1.31 | 1.6 | 1.41 | 1.56 | 1.25 | 1.56 |
|  | comments | OKAY | OKAY | OKAY | OKAY | OKAY | OKAY | C | OKAY | OKAY | OKAY | OKAY | OKAY |
| F | Genes | PRC1 | PRKCA | PTEN | PTTG1 | RB1 | RELA | RPRM | SESN2 | SIAH1 | SIRT1 | STAT1 | TADA3 |
|  | folds | 1.37 | 1.4 | 1.55 | 1.68 | 1.25 | 1.65 | 1.75 | 1.33 | 1.31 | 1.13 | 1.98 | 2.1 |
|  | comments | OKAY | OKAY | OKAY | OKAY | OKAY | OKAY | A | OKAY | OKAY | OKAY | OKAY | OKAY |
| G | Genes | TNF | TNFRSF10B | TNFRSF10D | TP53 | TP53AIP1 | TP53BP2 | TP63 | TP73 | TRAF2 | TSC1 | WT1 | XRCC5 |
|  | folds | -1.71 | 1.06 | 1.37 | 1.7 | -1.31 | 1.72 | 5.5 | 2.81 | 1.09 | 1.62 | -1.31 | 2.3 |
|  | comments | B | OKAY | OKAY | OKAY | C | OKAY | A | A | OKAY | OKAY | C | OKAY |

**Analysis results of Signal Transduction**

|  | Layout | 1 | 2 | 3 | 4 | 5 | 6 | 7 | 8 | 9 | 10 | 11 | 12 |
| --- | --- | --- | --- | --- | --- | --- | --- | --- | --- | --- | --- | --- | --- |
| A | Genes | ACSL3 | ACSL4 | ACSL5 | ADM | ARNT | ATF4 | AXIN2 | BAX | BBC3 | BCL2 | BCL2A1 | BCL2L1 |
|  | folds | 1.21 | 1.07 | 3.64 | -1.48 | -1.41 | -1.26 | 3.1 | 2.09 | -1.01 | 1.06 | -1.26 | 1.6 |
|  | comments | OKAY | OKAY | A | OKAY | OKAY | OKAY | B | OKAY | OKAY | OKAY | B | OKAY |
| B | Genes | BIRC3 | BMP2 | BMP4 | BTG2 | CA9 | CCL5 | CCND1 | CCND2 | CDKN1A | CDKN1B | CEBPD | CPT2 |
|  | folds | -1.58 | -1.1 | -1.35 | 1.32 | -85.81 | 4.04 | 1.44 | -1.84 | 1.11 | -1.16 | 2.33 | 2.02 |
|  | comments | OKAY | OKAY | B | A | A | A | OKAY | C | OKAY | OKAY | OKAY | OKAY |
| C | Genes | CSF1 | DAB2 | EGFR | EMP1 | EPO | FABP1 | FAS | FCER2 | FOSL1 | FTH1 | GADD45A | GADD45B |
|  | folds | -1.01 | 1.9 | -1.1 | 2 | -3.83 | 1.81 | 1.34 | -1.48 | -1.7 | 1.96 | 2.17 | -1.75 |
|  | comments | OKAY | OKAY | OKAY | OKAY | B | B | OKAY | B | OKAY | OKAY | OKAY | OKAY |
| D | Genes | GATA3 | GCLC | GCLM | GSR | HERPUD1 | HES1 | HES5 | HEY1 | HEY2 | HEYL | HMOX1 | ICAM1 |
|  | folds | -1.5 | 1.69 | 2.43 | 1.78 | -1.07 | -1.83 | 1.18 | 1.48 | 1.55 | 1.36 | 3.04 | -1.27 |
|  | comments | B | OKAY | OKAY | OKAY | OKAY | OKAY | OKAY | OKAY | OKAY | B | OKAY | OKAY |
| E | Genes | ID1 | IFNG | IFRD1 | IRF1 | JAG1 | LDHA | LFNG | LRG1 | MCL1 | MMP7 | MYC | NOTCH1 |
|  | folds | -2.23 | -1.84 | -1.73 | 1.41 | -2.2 | 1.5 | 1.02 | 1.85 | -1.03 | 8.7 | -1.96 | 1.86 |
|  | comments | OKAY | C | OKAY | OKAY | OKAY | OKAY | OKAY | OKAY | OKAY | A | OKAY | OKAY |
| F | Genes | NQO1 | OLR1 | PCNA | PPARD | PTCH1 | RB1 | SERPINE1 | SLC27A4 | SLC2A1 | SOCS3 | SORBS1 | SQSTM1 |
|  | folds | 3.1 | 2.15 | 1.5 | 1.52 | 1.18 | 1.26 | -1.74 | 2.27 | 1.56 | -1.26 | 1.21 | 1.4 |
|  | comments | OKAY | OKAY | OKAY | OKAY | OKAY | OKAY | OKAY | OKAY | OKAY | OKAY | OKAY | OKAY |
| G | Genes | STAT1 | TNF | TNFSF10 | TXN | TXNRD1 | VEGFA | WISP1 | WNT1 | WNT2B | WNT3A | WNT5A | WNT6 |
|  | folds | 1.74 | -4.76 | 2.45 | 2.66 | 1.64 | -1.62 | -3.53 | -1.61 | 1.64 | 1.71 | 1.07 | 1.75 |
|  | comments | OKAY | B | B | OKAY | OKAY | OKAY | B | B | OKAY | B | OKAY | OKAY |

**Analysis results of Tumor Metastasis PCR array**

|  | Layout | 1 | 2 | 3 | 4 | 5 | 6 | 7 | 8 | 9 | 10 | 11 | 12 |
| --- | --- | --- | --- | --- | --- | --- | --- | --- | --- | --- | --- | --- | --- |
| A | Genes | APC | BRMS1 | CCL7 | CD44 | CD82 | CDH1 | CDH11 | CDH6 | CDKN2A | CHD4 | COL4A2 | CST7 |
|  | folds | 1.62 | 1.41 | -1.56 | -1.37 | 1.45 | 1.55 | 1.06 | 1.06 | 1.17 | 1.46 | -1.62 | 1.79 |
|  | comments | OKAY | OKAY | B | OKAY | OKAY | OKAY | C | C | OKAY | OKAY | OKAY | B |
| B | Genes | CTBP1 | CTNNA1 | CTSK | CTSL | CXCL12 | CXCR2 | CXCR4 | DENR | EPHB2 | ETV4 | EWSR1 | FAT1 |
|  | folds | 1.29 | 1.37 | -1.1 | 1.19 | 1.06 | -4.11 | -2.87 | 1.58 | 1.43 | -3.02 | 1.4 | 1.15 |
|  | comments | OKAY | OKAY | OKAY | OKAY | C | B | OKAY | OKAY | OKAY | OKAY | OKAY | OKAY |
| C | Genes | FGFR4 | FLT4 | FN1 | FXYD5 | GNRH1 | HGF | HPSE | HRAS | HTATIP2 | IGF1 | IL18 | IL1B |
|  | folds | 1.68 | 1.06 | -1.45 | -1.03 | -1.68 | -1.39 | 1.75 | 2.7 | 1.48 | -1.94 | -1.61 | -3.44 |
|  | comments | OKAY | C | OKAY | OKAY | OKAY | B | OKAY | OKAY | OKAY | B | OKAY | A |
| D | Genes | ITGA7 | ITGB3 | KISS1 | KISS1R | KRAS | MCAM | MDM2 | MET | METAP2 | MGAT5 | MMP10 | MMP11 |
|  | folds | 1.01 | -1.05 | -1.99 | -1 | 1.2 | -1.34 | 1.16 | -1.12 | -1.04 | -1.22 | -2.18 | 2.16 |
|  | comments | OKAY | B | B | B | OKAY | OKAY | OKAY | OKAY | OKAY | OKAY | B | A |
| E | Genes | MMP13 | MMP2 | MMP3 | MMP7 | MMP9 | MTA1 | MTSS1 | MYC | MYCL | NF2 | NME1 | NME4 |
|  | folds | 1.45 | 18.15 | 1.06 | 5.94 | -1.07 | -1.21 | 2.1 | -2.27 | -1.13 | 1.16 | 1.89 | 1.2 |
|  | comments | B | B | C | OKAY | OKAY | OKAY | OKAY | OKAY | B | OKAY | OKAY | OKAY |
| F | Genes | NR4A3 | PLAUR | PNN | PTEN | RB1 | RORB | RPSA | SERPINE1 | SET | SMAD2 | SMAD4 | SRC |
|  | folds | -2.29 | -1.07 | -1.5 | 1.21 | 1.03 | -1.36 | 1.4 | -1.78 | -1.1 | -1.32 | -1.24 | -1.01 |
|  | comments | OKAY | OKAY | OKAY | OKAY | OKAY | OKAY | OKAY | OKAY | OKAY | OKAY | OKAY | OKAY |
| G | Genes | SSTR2 | SYK | TCF20 | TGFB1 | TIMP2 | TIMP3 | TIMP4 | TNFSF10 | TP53 | TRPM1 | TSHR | VEGFA |
|  | folds | -3.33 | 1.06 | -1.03 | -1.34 | 1.03 | -1.98 | 2.15 | 1.41 | -1.06 | 1.06 | 1.06 | -1.98 |
|  | comments | B | C | OKAY | OKAY | OKAY | OKAY | OKAY | B | OKAY | C | C | OKAY |
