# Supplementary material for: PADI4 has genetic susceptibility to gastric carcinoma and upregulates CXCR2, KRT14 and TNF-α expression levels
Source: Oncotarget. 2016 Aug 19;7(38):62159–76. doi: 10.18632/oncotarget.11398 (PMC5308718; doi:10.18632/oncotarget.11398)
Supplement: Supplementary file 3 [file oncotarget-07-62159-s003.docx]

**Additional file 1. SNP information**

| **SNP** | **Gene_Symbol** | **Chr. Location** | **Gene Location** |
| --- | --- | --- | --- |
| rs35381732 | PADI4 | 17634740 | CODING |
| rs78185114 | PADI4 | 17634831 | INTRON |
| rs2501798 | PADI4 | 17635036 | INTRON |
| rs1886301 | PADI4 | 17635411 | INTRON |
| rs1635598 | PADI4 | 17657321 | INTRON |
| rs77182432 | PADI4 | 17657536 | CODING |
| rs11203367 | PADI4 | 17657616 | CODING |
| rs77519194 | PADI4 | 17657779 | INTRON |
| rs72916857 | PADI4 | 17657852 | INTRON |
| rs35809798 | PADI4 | 17657986 | INTRON |
| rs882537 | PADI4 | 17660047 | INTRON |
| rs78540609 | PADI4 | 17660352 | INTRON |
| rs874881 | PADI4 | 17660499 | CODING |
| rs57744451 | PADI4 | 17660601 | INTRON |
| rs1748034 | PADI4 | 17662541 | INTRON |
| rs2240340 | PADI4 | 17662639 | INTRON |
| rs12733102 | PADI4 | 17662705 | CODING |
| rs1748032 | PADI4 | 17662804 | INTRON |
| rs11587622 | PADI4 | 17664257 | INTRON |
| rs12089685 | PADI4 | 17664480 | INTRON |
| rs11588132 | PADI4 | 17664615 | CODING |
| rs1635586 | PADI4 | 17664770 | INTRON |
| rs1635584 | PADI4 | 17664836 | INTRON |
| rs11203368 | PADI4 | 17666508 | INTRON |
| rs77381424 | PADI4 | 17668160 | INTRON |
| rs1748021 | PADI4 | 17668270 | INTRON |
| rs72916895 | PADI4 | 17668395 | INTRON |
| rs16825565 | PADI4 | 17668508 | CODING |
| rs1748020 | PADI4 | 17668609 | CODING |
| rs76761936 | PADI4 | 17668788 | INTRON |
| rs33981382 | PADI4 | 17668888 | CODING |
| rs1748019 | PADI4 | 17668976 | INTRON |
| rs6662651 | PADI4 | 17669911 | INTRON |
| rs77549925 | PADI4 | 17669975 | INTRON |
| rs2240337 | PADI4 | 17674222 | INTRON |
| rs75423317 | PADI4 | 17674298 | INTRON |
| rs2240336 | PADI4 | 17674402 | INTRON |
| rs2240335 | PADI4 | 17674537 | CODING |
| rs1635570 | PADI4 | 17674601 | INTRON |
| rs12746252 | PADI4 | 17674717 | INTRON |
| rs12746451 | PADI4 | 17674821 | INTRON |
| rs76384086 | PADI4 | 17680587 | INTRON |
| rs76612977 | PADI4 | 17680746 | INTRON |
| rs4920599 | PADI4 | 17680845 | INTRON |
| rs35240185 | PADI4 | 17681144 | CODING |
| rs6683201 | PADI4 | 17681277 | INTRON |
| rs79100767 | PADI4 | 17681339 | INTRON |
| rs4920600 | PADI4 | 17681424 | INTRON |
| rs74452983 | PADI4 | 17681596 | INTRON |
| rs10437048 | PADI4 | 17682719 | INTRON |
| rs2301887 | PADI4 | 17682923 | CODING |
| rs1635567 | PADI4 | 17683041 | INTRON |
| rs1635566 | PADI4 | 17683157 | INTRON |
| rs1748011 | PADI4 | 17683281 | INTRON |
| rs1635564 | PADI4 | 17683526 | INTRON |
| rs1635561 | PADI4 | 17687027 | INTRON |
| rs2477150 | PADI4 | 17689954 | INTRON |
| rs77757441 | PADI4 | 17690276 | UTR |
| rs34954650 | PADI4 | 17690376 | UTR |
